# A novel concurrent TMS‐fMRI method to reveal propagation patterns of prefrontal magnetic brain stimulation

**DOI:** 10.1002/hbm.24307

**Published:** 2018-08-29

**Authors:** Jord J. T. Vink, Stefano Mandija, Petar I. Petrov, Cornells A. T. van den Berg, Iris E. C. Sommer, Sebastiaan F. W. Neggers

**Affiliations:** ^1^ Department of Imaging University Medical Center Utrecht, Utrecht University Utrecht The Netherlands; ^2^ Department of Psychiatry University Medical Center Utrecht, Utrecht University Utrecht The Netherlands; ^3^ Department of Neuroscience and Psychiatry University Medical Center Groningen, University of Groningen Groningen The Netherlands; ^4^ Department of Medical and Biological Psychology University of Bergen Bergen Norway

**Keywords:** concurrent TMS‐fMRI, depression, dorsolateral prefrontal cortex, functional MRI, major depressive disorder, subgenual anterior cingulate cortex, transcranial magnetic stimulation

## Abstract

Major depressive disorder (MDD) is a severe mental disorder associated with high morbidity and mortality rates, which remains difficult to treat, as both resistance and recurrence rates are high. Repetitive transcranial magnetic stimulation (TMS) of the left dorsolateral prefrontal cortex (DLPFC) provides a safe and effective treatment for selected patients with treatment‐resistant MDD. Little is known about the mechanisms of action of TMS provided to the left DLPFC in MDD and we can currently not predict who will respond to this type of treatment, precluding effective patient selection. In order to shed some light on the mechanism of action, we applied single pulse TMS to the left DLPFC in 10 healthy participants using a unique TMS‐fMRI set‐up, in which we could record the direct effects of TMS. Stimulation of the DLPFC triggered activity in a number of connected brain regions, including the subgenual anterior cingulate cortex (sgACC) in four out of nine participants. The sgACC is of particular interest, because normalization of activity in this region has been associated with relief of depressive symptoms in MDD patients. This is the first direct evidence that TMS pulses delivered to the DLPFC can propagate to the sgACC. The propagation of TMS‐induced activity from the DLPFC to sgACC may be an accurate biomarker for rTMS efficacy. Further research is required to determine whether this method can contribute to the selection of patients with treatment resistant MDD who will respond to rTMS treatment.

**Abbreviations**
COG: Center of gravity
DBS: Deep brain stimulation
DLPFC: Dorsolateral prefrontal cortex
ECT: Electroconvulsive therapy
EPI: Echo‐planar imaging
MDD: Major depressive disorder
sgACC: Subgenual anterior cingulate cortex
TR‐MDD: Treatment resistant MDD

## 1 INTRODUCTION

Major depressive disorder (MDD) is a complex disorder characterized by a depressed mood and/or loss of interest or pleasure in (almost) all activities (American Psychiatric Association, 2013). It affects 4.7% of the global population and is the second leading cause of disability worldwide (Ferrari, Charlson, et al., 2013; Ferrari, Somerville, et al., 2013). MDD is currently treated by means of antidepressant medication, psychotherapy (often behavioral therapy) or a combination of these two. Treatment resistant patients are treated with electro‐convulsive therapy or, in rare cases, with deep brain stimulation. Both psychotherapy and pharmacotherapy have small effect sizes of around 0.3, leaving a substantial amount of patients without adequate therapy due to intolerance or unresponsiveness (Cuijpers, Cristea, Karyotaki, Reijnders, & Hollon, 2017; Little, 2009; Locher et al., 2017). Up to two thirds of the patients with MDD do not respond to the first medication prescribed and approximately 15–33% of patients suffer from treatment resistance, which is defined as unresponsiveness or intolerance to at least two different classes of antidepressants (Cain, 2007). This stresses the need for more treatment options for patients with treatment‐resistant major depressive disorder (TR‐MDD). Repetitive transcranial magnetic stimulation (rTMS) and electroconvulsive therapy (ECT) provide treatment alternatives for patients with TR‐MDD. Unfortunately, ECT has major disadvantages, including the need for anesthesia and severe side effects like amnesia (Ingram, Saling, & Schweitzer, 2008).

Unlike ECT, rTMS is a targeted noninvasive brain stimulation method with only mild side effects and has proven to be effective in the treatment of TR‐MDD (George, Lisanby, Avery, Mcdonald, & Durkalski, 2014). TMS is a means of using electromagnetic induction in order to stimulate a brain region. Repetitive delivery of TMS pulses (rTMS) to a brain region modulates the excitability of the stimulated area, inducing changes in neural plasticity (Allen, Pasley, Duong, & Freeman, 2007). These neuroplastic changes outlast the duration of stimulation and are believed to be induced through long‐term potentiation/depression mechanisms (Esser et al., 2006; Ishikawa et al., 2007). High (>5 Hz) or low (<5 Hz) frequency stimulation results in a lasting increase or decrease in excitability, respectively (Esser et al., 2006; Valero‐Cabré, Payne, & Pascual‐Leone, 2007). Stimulation is applied to a focal region in the brain, but the effects of TMS are not limited to the stimulated brain region, but spread to other cortical areas (Bestmann, Baudewig, Siebner, Rothwell, & Frahm, 2005; De Weijer et al., 2014; Rahman et al., 2013). Repetitive stimulation has been shown to induce a clinically relevant effect in MDD and has recently obtained FDA approval for its application in MDD (George et al., 2014; O'Reardon et al., 2007). Patients with MDD are treated through high frequency stimulation of the dorsolateral prefrontal cortex (DLPFC), which causes an antidepressant effect in around 25% of patients with TR‐MDD (O'Reardon et al., 2007).

Although already applied clinically, little is known about the mechanism of action of high frequency stimulation of the DLPFC. Over the last decades neuroimaging studies have investigated changes in the brain related to MDD intensively and several neuroanatomical regions have been found to exhibit abnormal activity in patients with MDD, with the subgenual anterior cingulate cortex (sgACC) attracting most attention. Neuroimaging studies have shown that baseline metabolic activity in the sgACC is increased in patients with MDD and that normalization of the activity in the sgACC correlates with relief of depressive symptoms (Kennedy et al., 2001; Mayberg et al., 2000, 2005; Videbech, 2000). It is hypothesized that rTMS of the DLPFC induces an antidepressant effect through direct or indirect neuromodulation of the abnormal activity in the sgACC (Fox, Buckner, White, Greicius, & Pascual‐Leone, 2012). MRI functional connectivity studies show that treatment outcome positively correlates with functional connectivity strength between the sgACC and the DLPFC, providing some evidence for this hypothesis (Baeken et al., 2014). However, the evidence is limited as only a small number of patients received rTMS treatment. Furthermore, Fox and Baeken assume that a functional connection based on resting state fMRI data provides the pathway for TMS effects evoked in the DLPFC, while there is no evidence that shows that TMS‐induced activity can actually propagate from the DLPFC to the sgACC.

The antidepressant effect of rTMS is restricted to a quarter of the patients with TR‐MDD and is known to vary substantially between patients (O'Reardon et al., 2007). This stresses the need for a better understanding of the effects of rTMS treatment of the DLPFC in order to identify the patients who will respond to this type of rTMS treatment. Identification of the individual propagation pattern of TMS‐evoked activity in response to stimulation of the left DLPFC can reveal the mechanisms of action of rTMS treatment and provide clues for further improvement of such treatment using individualized treatment methods. This can be done by using a patient's individual response to TMS to optimize the effects after subsequent treatment with repetitive stimulation. More specifically, identification of the propagation patterns can reveal whether TMS‐induced activity evoked at the DLPFC has the ability to propagate to the sgACC, and potentially modulate the activity in the sgACC (Fox et al., 2012).

Therefore, we investigated the propagation pattern of TMS‐induced activity after stimulation of the left DLPFC using single pulse TMS. Because TMS effects are strongly affected by TMS coil placement (with respect to individual brain morphology), we also investigated the effect of TMS coil placement on propagation patterns of TMS‐induced activity (Fitzgerald et al., 2009; Sack et al., 2009). In order to achieve these goals, we applied single pulses of TMS to the left DLPFC during a functional MRI recording in 10 healthy participants, using a novel concurrent TMS‐fMRI technique (De Weijer et al., 2014).

## 2 MATERIALS AND METHODS

The experimental procedure was approved by the medical ethical committee of the University Medical Center Utrecht (UMCU), Utrecht, The Netherlands. All participants provided written informed consent and were screened for MRI and TMS exclusion criteria. MRI data were acquired in 10 right‐handed participants (Table 1). One participant had to be excluded due to unavailability during the follow‐up session. During the experimental procedure, we strictly adhered to the guidelines and recommendations for TMS endorsed by the International Federation for Clinical Neurophysiology (Rossi et al., 2009).

**Table 1:** Participant details

Participantnumber	Sex	Age	RMT	Comment	COG of TMS area			Max displacementfrom the COG (mm)	TMS‐MRI of M1
					*X*	*Y*	*Z*		
1	F	21	66		−43	23	45	4.4	Yes
2	M	34	73		−25	18	62	3.3	No
3	F	25	76	Excluded	–	–	–	–	–
4	M	18	58		−29	41	37	6.1	No
5	F	19	83		−33	25	47	5.2	Yes
6	F	24	83		−30	33	41	5.0	Yes
7	M	23	80		−29	28	52	2.1	Yes
8	F	20	78		−35	25	47	3.6	Yes
9	F	19	83		−33	22	57	2.9	Yes
10	F	20	82		−30	14	51	2.5	No

*Notes*. The MNI coordinates of the normalized COG of the TMS area are shown for each participant. The TMS area is based on initial TMS coil placement and corrected for subsequent head motion during image acquisition. The maximum displacement of the TMS target from the COG reflects the effect of head movement on the displacement of the TMS coil isocenter.

Our concurrent TMS‐fMRI setup has previously been used to successfully detect TMS‐induced activity in the motor network in response to TMS pulses delivered to the primary motor cortex (M1) of healthy participants (De Weijer et al., 2014). We used this setup to stimulate M1 again, but primarily investigated TMS‐induced activity in response to stimulation of the left DLPFC of healthy participants. Both experiments were done using a biphasic Magstim Rapid^2^ magnetic stimulator, an MR‐compatible TMS coil and a custom designed TMS filter box (all manufactured by Magstim Inc., Whitland, The United Kingdom, http://www.magstim.com; De Weijer et al., 2014; Mandija, Petrov, Neggers, Luijten, & Berg, 2016). All MR sequences (MRI‐only and concurrent TMS‐MRI) were performed in a 3T MR scanner (Achieva, Philips Healthcare, Best, The Netherlands).

### 2.1 Data acquisition

**Figure 1:**
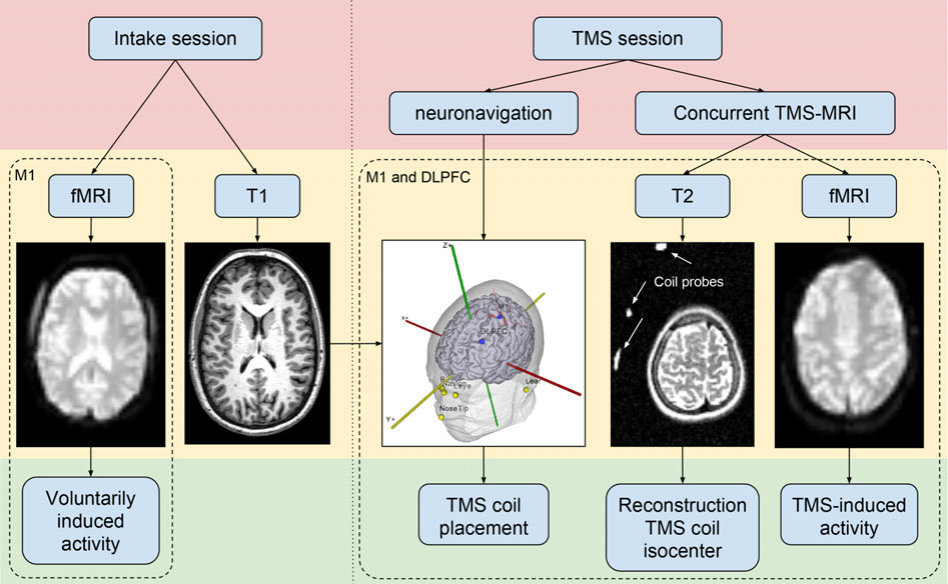
A flowchart of the experimental procedure. The fMRI scan during the intake was used in order to compare voluntarily induced motor network activity with TMS‐induced activity. The concurrent TMS‐fMRI session consisted of two subsections: An M1 and a DLPFC subsection

The experiment was divided into two parts: an intake session (MRI‐only) and a TMS session (concurrent TMS‐MRI) (Figure 1). These sessions are described below.

#### 2.1.1 Intake session

First, a 3D T1 weighted anatomical scan was acquired with a TR/TE of 10.0/4.6 ms, a flip angle of 8°, voxel size of 0.75 × 0.75 × 0.8 mm^3^, scan duration of 11.3 min, 225 slices with a slice gap of 0 mm. This scan was used for neuronavigation during the TMS session and other visualization purposes.

Next, a single‐shot echo‐planar imaging (EPI) scan was acquired with 250 dynamics, a TR/TE of 2,000.0/23.0 ms, flip angle of 70°, voxel size of 4 × 4 × 4 mm^3^, a scan duration of 8.5 min and 30 slices with a slice thickness of 3.6 mm and a slice gap of 0.4 mm. During this scan, participants were instructed to move the thumb of the right hand upon presentation of an auditory cue. This scan was used to validate our concurrent TMS‐fMRI setup by comparing voluntarily‐induced motor network activity with TMS‐induced network activity in response to TMS of the primary motor cortex. Preprocessing and statistical analysis are described in the data analysis section.

#### 2.1.2 TMS session

**Figure 2:**
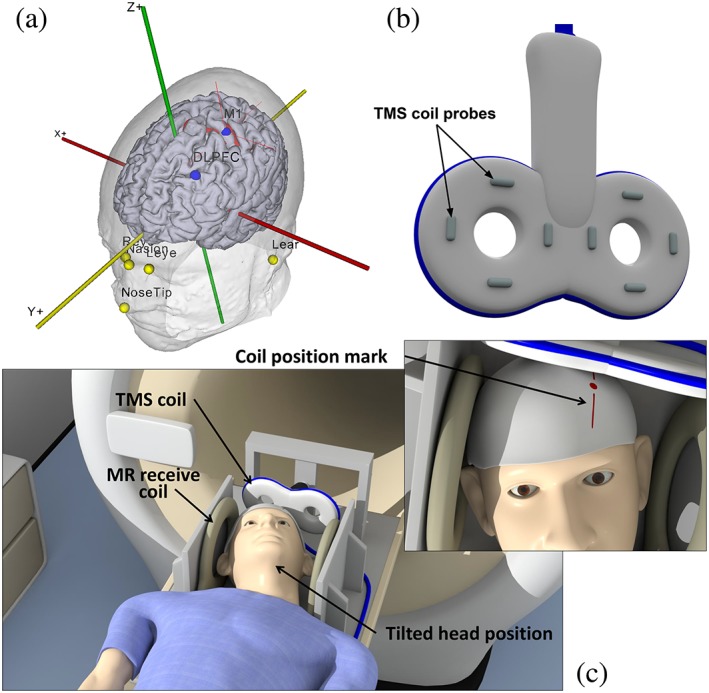
(a) Location of facial markers and TMS targets in the neural navigator. The statistical map of voluntarily‐induced motor activity is thresholded and shown in red. Facial markers: Tip of the nose; nasion, left and right inner eyelid; left and right upper and lower ear. TMS targets: Primary motor cortex (M1); dorsolateral prefrontal cortex (DLPFC). (b) TMS coil probes. The probes are visualized in a T2‐weighted scan to determine their location with respect to the head. (c) Participant is lying on the MR bed with the head positioned in between two MR receive coils and the TMS coil located over the cranium [Color figure can be viewed at http://wileyonlinelibrary.com]

For each participant, the T1 weighted image acquired during the intake session was segmented with SPM12 to obtain skin, skull, cerebrospinal fluid (CSF), white matter, and gray matter (GM) masks (Penny, Friston, Ashburner, Kiebel, & Nichols, 2011). The segmentations were used to visualize the 3D brain and skin surface in the Neural Navigator (Brain Science Tools, The Netherlands, http://www.neuralnavigator.com). The location of M1 was obtained from the statistical map acquired during the intake session and marked on the 3D brain surface. The TMS coil was oriented with the TMS coil handle perpendicular to the orientation of the precentral gyrus (at an angle of 30–45° to the midline depending on individual morphology) and pointing in posterior direction. The DLPFC target was placed three gyri (i.e., 3 cm) anterior to the premotor gyrus within the middle frontal gyrus, corresponding to the border between Brodmann areas 46 and 9 (Ahdab, Ayache, Brugières, Goujon, & Lefaucheur, 2010). For the DLPFC, the TMS coil handle was oriented perpendicular to the orientation of the middle frontal gyrus with the handle at 90° with the midline. Eight facial markers were used to align world space with the MRI coordinates: the upper and lower left and right ear, the left and right inner eye lid, the tip of the nose and the nasion (Figure 2a). Neuronavigation was then used to determine the TMS coil position for stimulation of M1. Markings were made on the bathing cap in order to be able to replicate the TMS coil position inside the scanner, since neuronavigation could not be performed inside the MRI scanner room. These markings were also made for the DLPFC.

Next, the TMS coil was placed over the left M1 guided by neuronavigation to determine the resting motor threshold (RMT). This was done by applying single pulses of TMS (with an inter‐stimulus interval of 7 s) to the primary motor cortex while increasing the TMS stimulator output until a response in the APB muscle was visible in 5 out of 10 TMS pulses (Schutter & van Honk, 2006).

The concurrent TMS‐MRI session was divided in two parts: In the first part TMS was applied to M1 and in the other part TMS was applied to the DLPFC. For the concurrent TMS‐MRI experiments, a custom made setup was used. The head was positioned in a custom designed setup between two MR receive coils (Figure 2c). The TMS coil was attached to a custom made mount which was positioned over the participant's cranium, eliminating potential TMS coil movement. Additionally, to minimize Lorentz forces on the TMS coil wings, the angle between the TMS coil plane and the MRI static magnetic field was limited to 25°. The head was tilted backward and rotated slightly to match the coil position with the markings on the bathing cap. The head and neck of the participant were supported to increase comfort and to minimize head movement during the scan.

After TMS coil positioning, two sequences were acquired. First, a T2‐weighted scan with a TR/TE of 13,609.0/80.0 ms, flip angle of 90°, voxel size of 2 × 2 × 2 mm^3^, scan duration of 3.6 min. The purpose of this scan was to visualize the coil location and orientation with respect to the head. This was done by attaching six custom made markers (small capsules filled with water) to the back of the TMS coil (Figure 2b), which appear hyper intense on the T2‐weighted scan (Figure 1). Second, a single‐shot EPI sequence was acquired with 500 dynamics, a TR/TE of 2,000.0/23.0 ms, flip angle of 70°, FOV of 256 × 119.6 × 208 mm^3^, matrix of 64 × 63, voxel size of 4 × 4 × 4 mm^3^, scan duration of 17 min, 30 slices with a slice thickness of 3.6 mm, and a slice gap of 0.4 mm. We acquired 500 dynamics to make sure that we had sufficient power to detect the effects of single pulses of TMS. During the EPI sequence, single pulses of TMS with an intensity of 115% RMT were interleaved with pulses with an intensity of 60% RMT. TMS pulses were delivered with a random interval of 5–8 dynamics (10–16 s) to avoid habituation.

### 2.2 Data analysis

The data obtained for stimulation of M1 and the DLPFC were analyzed in the same way. Analysis of the structural and fMRI data was performed with custom scripts and SPM12 (Penny et al., 2011) in the MATLAB R2014a environment (MathWorks Inc., Natik, MA, The United States).

The T1‐weighted image was segmented with SPM through unified segmentation with six tissue type priors to obtain a gray matter, white matter, and CSF mask (Ashburner & Friston, 2005).

All EPI volumes were inspected to determine image quality and to identify the presence of potential artifacts. This revealed small random deflections from the baseline signal level in a single slice of a few functional volumes per time series acquired during the TMS session. A small number of artifacts were present in most of the time series data. These deflections are short (one sample) and can only be observed in the vicinity of the TMS coil. All slices of the realigned EPI scans were automatically scanned for the presence of a sharp peak in the average gray matter signal with a custom algorithm to detect distortions. The distorted slices were then interpolated based on the BOLD signal in the previous and next slice with custom MATLAB code. About 15–70 slices were interpolated out of 30 slices and 500 volumes depending on the participant. These artifacts are discussed in more detail in the section “Image artifact.”

All EPI volumes were realigned and normalized using the nonlinear normalization parameters obtained from segmentation of the T1‐weighted scan. The EPI volumes were subsequently resliced at a resolution of 4 × 4 × 4 mm^3^ and smoothed with a FWHM of 8 mm.

The location of the TMS coil isocenter was reconstructed with respect to the brain based on the location of the fluid markers on the TMS coil. In order to reconstruct the TMS coil isocenter with respect to the brain, the T2‐weighted scan was first co‐registered to the anatomical T1‐weighted scan using SPM12. Then, based on the location of the TMS coil markers in the T2‐weighted scan, we were able to reconstruct the TMS coil position and isocenter, as described in De Weijer et al. (2014). Thereafter, the EPI volumes were realigned using SPM12 and the mean EPI scan was co‐registered to the T1‐weighted scan. The inverse of the EPI to T1 affine transformation and the inverse of the EPI realignment affine transformations were used to create a head movement‐corrected reconstruction of the location of the TMS coil isocenter. Thereafter, the center of gravity (COG) of the TMS coil isocenter was calculated by calculating the average of the 3D coordinates of the TMS coil isocenter over all volumes.

For the functional data obtained during the intake session, the thumb movements were modeled with the canonical hemodynamic response function (HRF) and its first‐order derivative in a standard event‐related GLM analysis with two nuisance regressors: the average BOLD signal in the white matter and the CSF. Statistical images were constructed based on an F‐statistic with the F‐threshold at *p* < .05, family wise error (FWE) corrected (Penny et al., 2011).

We performed subject‐level event‐related GLM analyses in SPM12. A group‐level analysis was not performed due to the limited sample size (Desmond & Glover, 2002; Thirion et al., 2007). The generalized linear model (GLM) included two events: single pulses of 115% RMT and 60% RMT. The BOLD response was modeled with the canonical HRF and its first‐order derivative. The mechanism through which TMS induces brain activity is different from conventional MRI tasks which investigate voluntary brain activity. Inclusion of the first‐order derivative allows more variability in the hemodynamic response, which allows more accurate modeling of the BOLD response during TMS pulse delivery. Two nuisance regressors were included in the analysis: the average BOLD signal in the white matter and the CSF. BOLD signals were filtered with a high pass filter of 80 Hz before construction of the GLM. Statistical images were constructed based on the contrast between TMS pulses of 115% RMT and TMS pulses of 60% RMT. An F‐statistic was used to test for variance explained by either the canonical HRF or the first‐order derivative as the sum of both weighted regressors in a first‐order Taylor expansion, using a threshold at *p* < .05, FWE corrected (Penny et al., 2011). TMS pulses of 115% RMT were contrasted with TMS pulses of 60% RMT to minimize the neural response to the sensation or sound that is accompanied by TMS pulse delivery.

## 3 RESULTS

### 3.1 Voluntary versus TMS‐induced motor activity (M1 session)

We compared motor network activity in response to voluntary hand movements with TMS‐induced activity after stimulation of M1. The full findings on TMS‐induced activity are summarized in Table 3 and all individual activation maps can be found in the Supporting Information.

**Figure 3:**
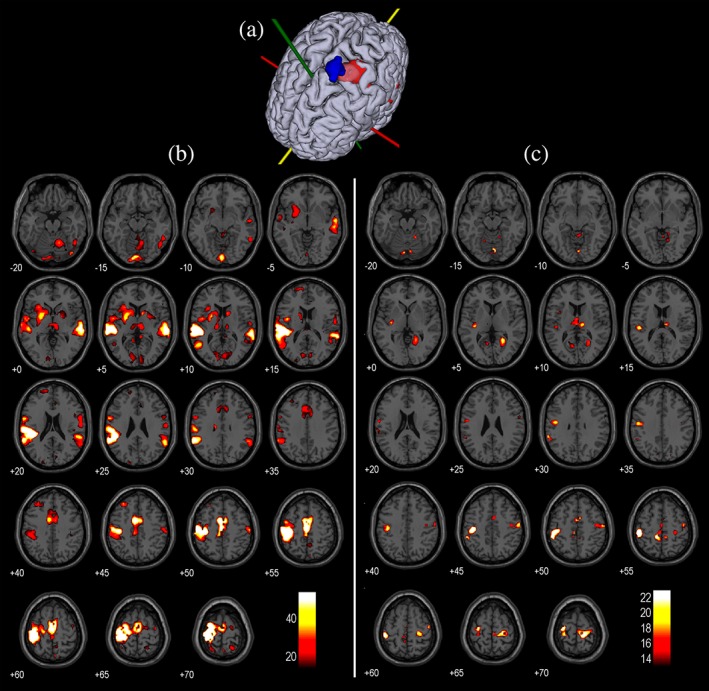
(a) Activity in the primary motor cortex in response to voluntary thumb movements (in red) and the location of the TMS coil isocenter during the TMS‐fMRI session is shown (in blue). (b) Activity in response to voluntary thumb movements contrasted with baseline activity (*p* < .05, FWE corrected). Axial slices of an MNI brain are shown (left = left). (c) TMS‐induced activity in response to TMS pulses of 115% RMT contrasted with baseline activity (*p* < .05, FWE corrected). The same slices are shown as in (b) [Color figure can be viewed at http://wileyonlinelibrary.com]

Observations are described in detail for Participant 1, who shows strong similarities between voluntarily‐induced brain activity and TMS‐induced activity. In this participant, voluntary movement of the right thumb results in observable activity in, among other regions, the left primary motor cortex (M1) and supplementary motor area (SMA), the right hemisphere of the cerebellum and the bilateral putamen and thalamus (*p* < .05, FWE corrected; Figure 3b). Because the participants were instructed to move their thumb based on auditory cues, activity can also be observed in the primary auditory cortex (A1).

During the concurrent TMS‐fMRI session, the TMS coil isocenter was located slightly medio‐anterior to the maximum BOLD response in the precentral gyrus, with limited displacement of the TMS coil isocenter due to head movement during the session (Figure 3a). TMS‐induced activity was defined as the contrast between TMS pulses of 115% RMT and 60% RMT. The participant reported thumb movement in response to high intensity TMS pulses throughout the majority of the session and reported no thumb movements for low intensity TMS pulses. TMS‐induced activity can be observed in the bilateral M1 and thalamus, the left SMA and putamen and the right hemisphere of the cerebellum (*p* < .05, FWE corrected; Figure 3c). Because TMS pulse delivery is accompanied by an auditory “click,” activity can also be observed in A1. Additionally, TMS‐induced activity can be observed in the bilateral primary visual cortices.

### 3.2 TMS target (DLPFC session)

**Figure 4:**
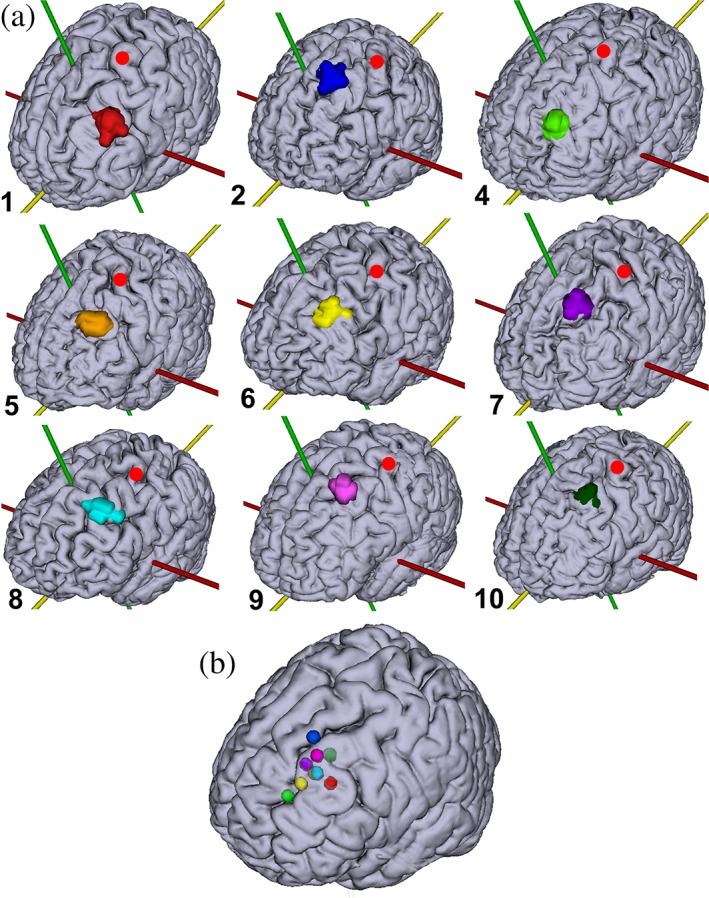
(a) TMS targets projected onto individual brain surfaces. The numbers refer to the participant numbers in Table 1. The red dot indicates the location of the hand area in the primary motor cortex (M1) as determined through fMRI. (b) The normalized COG of each individual TMS target projected onto an MNI brain. The TMS targets are based on initial TMS coil placement (as determined through a T2‐weighted scan) and corrected for subsequent head movement during image acquisition (as determined through realignment of the EPI volumes). Corresponding colors are used in (a) and (b) and Table 1 [Color figure can be viewed at http://wileyonlinelibrary.com]

The locations of the COG of the TMS coil isocenters were located well within the anatomical landmarks of the DLPFC in all participants (Figure 4a). The TMS coil isocenter remained within the DLPFC for the majority of the session in all participants, despite small head movements. Head movement resulted in a maximum displacement of the TMS coil isocenter from the COG of 2.1–6.1 mm (mean: 4 mm) depending on the participant (Table 1), which shows that TMS coil placement was accurate and head movement was limited. In the majority of the participants, the TMS coil isocenter was located in the posterior part of the DLPFC, while the TMS coil isocenter was located in the anterior part of the DLPFC for Participants 4 and 6. The normalized COGs of the TMS coil isocenter cluster in the left DLPFC (Figure 4b), with the COGs of Participants 2 and 4 located on the edges of the middle frontal gyrus.

### 3.3 TMS‐induced activity (DLPFC + M1 session)

An overview of neuroanatomical regions that show TMS‐induced activity in response to TMS pulses delivered to the left DLPFC (Table 2) and the left M1 (Table 3) are shown for all participants.

**Table 2:** Summary of TMS‐induced activity observed in individual participants

#	DLPFC	VLPFC	APFC	MPFC	PM	OFC	S1	sgACC	SPL	Temp
1	Left									
2	Left	Left	Left		Left					Right
3	–	–	–	–	–	–	–	–	–	–
4		Left			Left					
5	Left	Left	Left	Left	Left	Bi		Left		Right
6	Left	Left					Left			
7	Left	Left	Left	Left				Left		
8		Left					Left	Left		
9	Left	Left	Left	Left	Left		Left	Left		
10	Left								Left	Bi

DLPFC = dorsolateral prefrontal cortex; VLPFC = ventrolateral prefrontal cortex; APFC = anterior prefrontal cortex; MPFC = medial prefrontal cortex; PM = premotor cortex; OFC = orbitofrontal cortex; S1 = primary somatosensory cortex; sgACC = subgenual anterior cingulate cortex; SPL = superior parietal lobule; temp, temporal lobe.

**Table 3:** Summary of TMS‐induced activity after stimulation of M1 with 115% RMT contrasted with 60% RMT of individual participants

#	M1	PM	S1	SMA	Put	Thal	A1	Cer	Temp
1	Bi	Left	Left		Left				
2	–	–	–	–	–	–	–	–	–
3	–	–	–	–	–	–	–	–	–
4	–	–	–	–	–	–	–	–	–
5	Bi	Bi					Left		Left
6	Bi	Bi	Bi	Bi				Left	Bi
7	Bi	Bi	Right	Left	Left		Left	Left	Bi
8	Left	Right	Left					Left	
9	Bi	Bi	Bi	Bi	Bi			Left	Left
10	–	–	–	–	–	–	–	–	–

M1 = primary motor cortex; PM = premotor cortex; S1 = somatosensory cortex; SMA = supplementary motor area; put = putamen; Thal = thalamus; A1 = primary auditory cortex; Cer = cerebellum; temp = temporal lobe.

**Figure 5:**
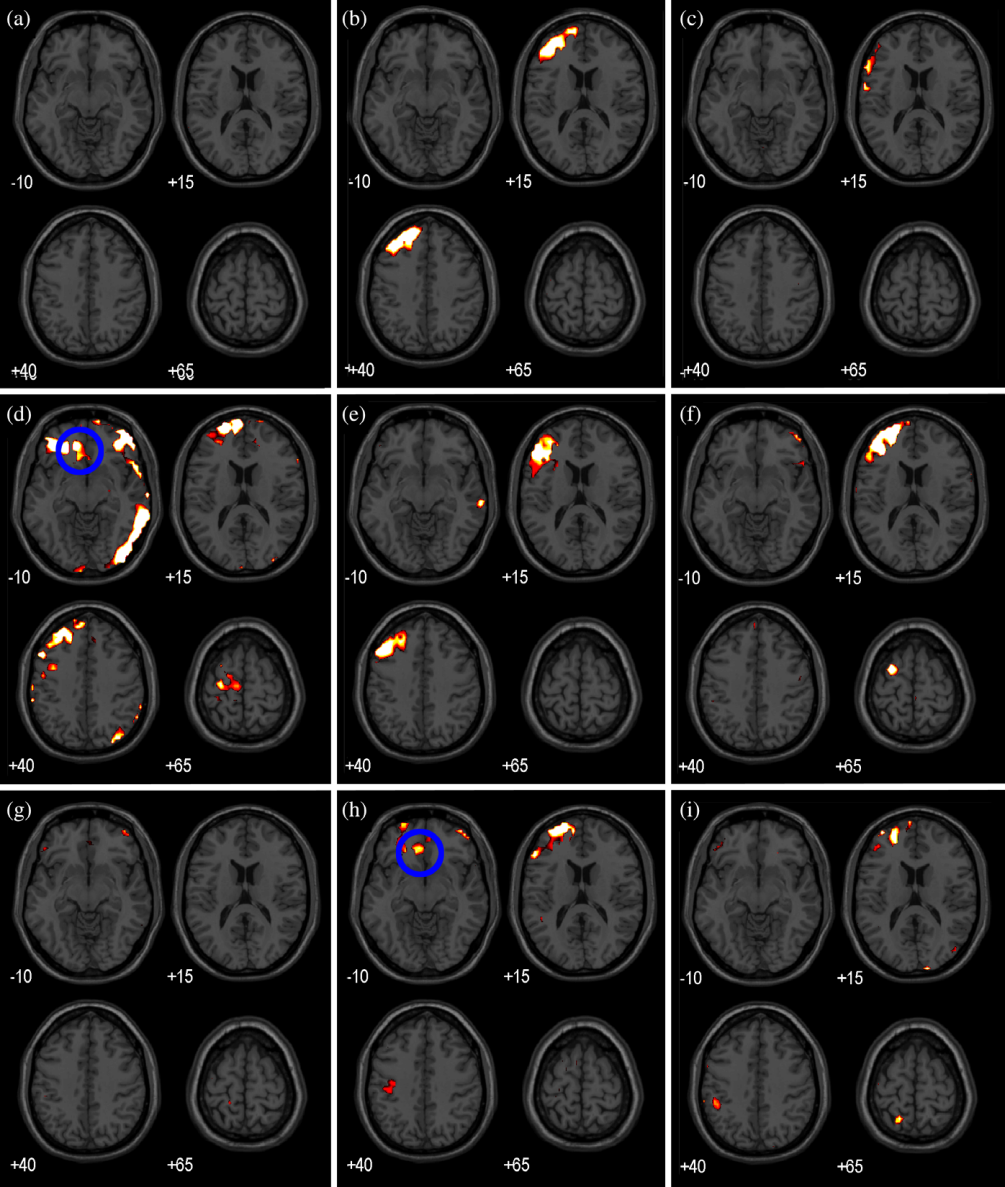
TMS‐induced activity in response to TMS pulses of 115% RMT contrasted with TMS pulses of 60% RMT (*p* < .05, FWE corrected). Axial slices of an MNI brain are shown (left = left). (a)–(i) show TMS‐induced activity of Participants 1, 2, and 4–10 [Color figure can be viewed at http://wileyonlinelibrary.com]

An overview of TMS‐induced activity in response to left DLPFC stimulation is shown on four slices of an MNI brain for each participant (Figure 5). These slices show activity in response to TMS pulses of 115% RMT contrasted with TMS pulses of 60% RMT (*p* < .05, FWE corrected). The observed propagation patterns of TMS‐induced activity show substantial variation between participants. However, all participants show TMS‐induced activity in one or more subdivisions of the left prefrontal cortex. Interestingly, TMS‐induced activity is generally absent in right hemisphere (contralateral to the stimulation site). Although TMS‐induced activity is predominantly present in the left prefrontal area, it does also propagate to distant areas: including parietal and temporal areas.

**Figure 6:**
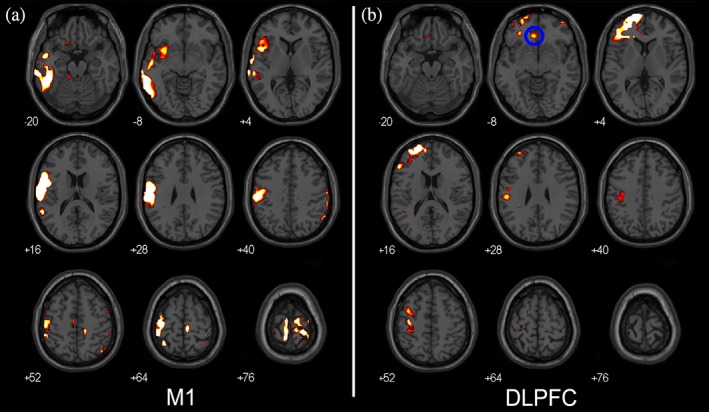
TMS‐induced activity in response to TMS pulses of 115% RMT contrasted with TMS pulses of 60% RMT (*p* < .05, FWE corrected). Axial slices of an MNI brain are shown (left = left). (a) and (b) show TMS‐induced activity in two different participants in which the TMS target was very similar. Left is left. (a) TMS‐induced activity of Participant 9. (b) TMS‐induced activity of Participant 10 [Color figure can be viewed at http://wileyonlinelibrary.com]

Figure 6 shows TMS‐induced activity evoked by stimulation of the left M1 and DLPFC of Participant 9. Stimulation of the left M1 reveals TMS‐induced activity in the bilateral M1 and premotor cortex, which is more abundant in the lateral parts (representation of the face). Activity can also be observed in left SMA. Application of TMS to the left DLPFC reveals activity in a large part of the left prefrontal cortex and a small cluster in the left M1 and S1.

**Figure 7:**
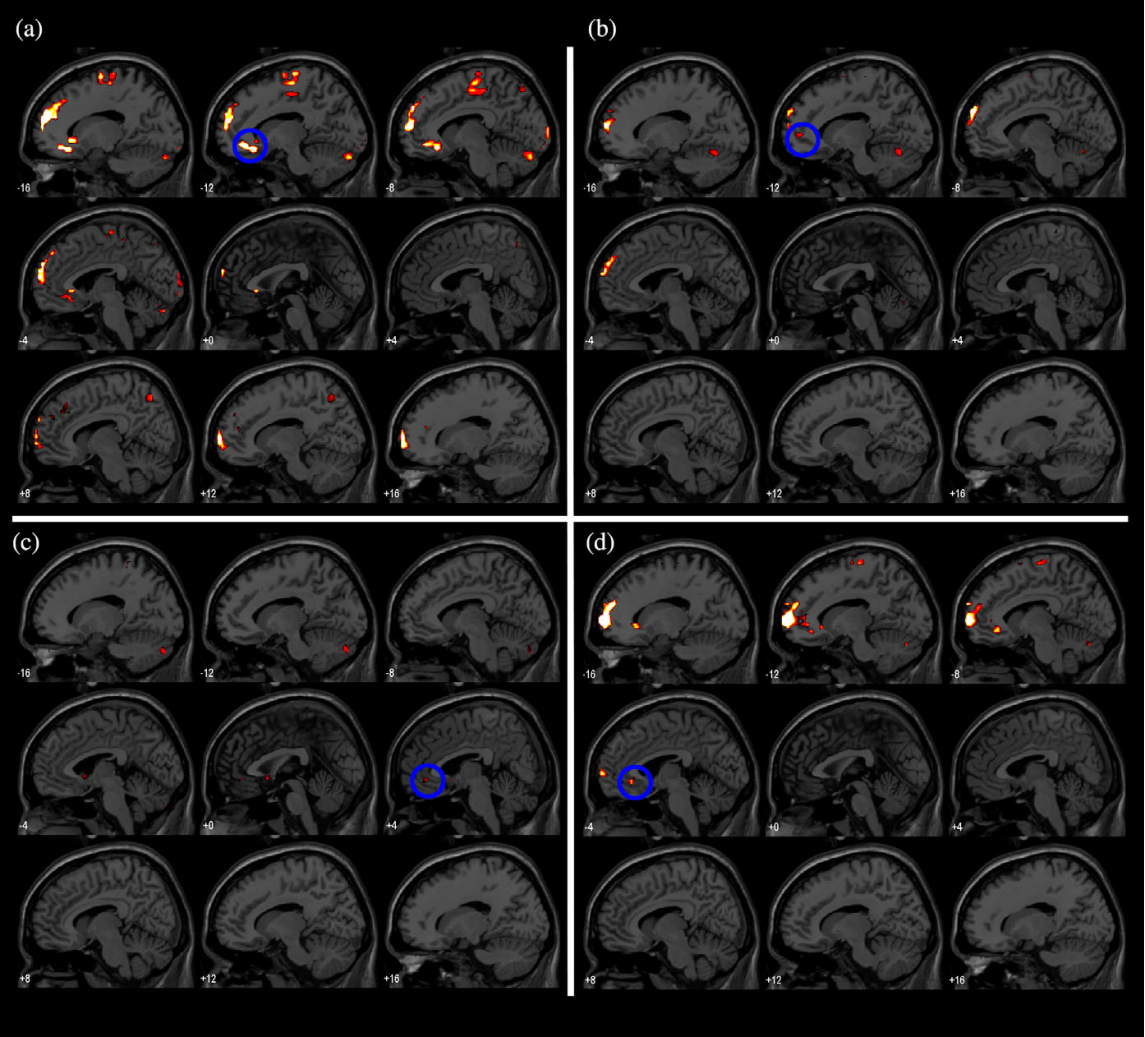
TMS‐induced activity in response to TMS pulses of 115% RMT contrasted with TMS pulses of 60% RMT (*p* < .05, FWE corrected). Sagittal slices of an MNI brain are shown (first slice = left side; last slice = right side). (a) TMS‐induced activity of Participant 5. (b) TMS‐induced activity of Participant 7. (c) TMS‐induced activity of Participant 8. (d) TMS‐induced activity of Participant 9 [Color figure can be viewed at http://wileyonlinelibrary.com]

Figure 7 shows TMS‐induced activity in the limbic region in response to DLPFC stimulation, specifically focusing on activity in the sgACC. The delivery of TMS pulses to the DLPFC causes activity in the sgACC in four out of nine participants, all of whom show activity in the left (ipsilateral to stimulation) sgACC (Table 2). However, Participant 7 shows TMS‐induced activity on the boundary between the subgenual ACC and the neighboring ACC. Activation of the sgACC is observed exclusively for stimulation of the DLPFC.

## 4 DISCUSSION

Using a novel concurrent TMS‐MRI setup, we investigated the propagation patterns of TMS‐induced activity after stimulation of the left DLPFC and left M1. We found that TMS delivered to the DLPFC results in activity in the sgACC in four out of nine participants, while TMS delivered to the left M1 does not result in sgACC activity. This indicates that TMS‐induced activity evoked by TMS of the left DLPFC has the ability to, directly or indirectly, propagate from the DLPFC to the sgACC. The existence of such a pathway was already suggested by others who investigated the mechanisms of action of rTMS of the left DLPFC in MDD by exploring resting state functional connectivity (Fox et al., 2012). That work showed that the strength of the functional connection between the DLPFC and the sgACC correlated positively with treatment outcome after rTMS, implying that rTMS utilizes this functional connection (Baeken et al., 2014). However, evidence for a direct structural connection between these regions was lacking. Our observations provide the first direct evidence that TMS‐induced activity can propagate to the sgACC (at least in some individuals). Combining our observations with prior literature, it becomes apparent that the modulatory effect of rTMS of the DLPFC can potentially, directly or indirectly, propagate to the sgACC, depending on individual structural connectivity. The sgACC is an important area for psychiatric treatment as deep brain stimulation (DBS) of this area has shown to have both antidepressant and anti‐compulsive effects (Conen, Matthews, Patel, Anton‐Rodriguez, & Talbot, 2017; De Ridder, Leong, Manning, Vanneste, & Glue, 2017; Dougherty et al., 2016; Kibleur et al., 2017; Tsolaki, Espinoza, & Pouratian, 2017).

As stated previously, the treatment response is related to the strength of the functional connection between the DLPFC and the sgACC, which indicates that rTMS treatment of the DLPFC induces an antidepressant effect through modulation of the activity in the sgACC (Baeken et al., 2014). If this is indeed the therapeutic effect of DLPFC rTMS, the variability in propagation patterns of TMS‐induced activity provides a potential explanation for the limited response rate of <30% of rTMS in the treatment of MDD (O'Reardon et al., 2007). The propagation of TMS‐induced activity from the DLPFC to the sgACC in individual patients with MDD could be a predictor of a response to rTMS treatment. Further research is required to better understand propagation patterns of TMS‐induced activity and the relationship with treatment outcome.

It is important to note that we validated our concurrent TMS‐MRI setup by stimulating M1 and comparing TMS‐induced network activity to voluntarily‐induced motor network activity (Figure 3). This was done by stimulating M1 because of the extensive literature on motor networks and the ability to directly determine adequate stimulation through observation of induced thumb movements. For M1 stimulation, we observed TMS‐induced activity in neuroanatomical regions which are strongly associated with motor activity, like M1, SMA, putamen, thalamus and cerebellar subregions, and most of those regions were also observed in other concurrent TMS‐fMRI studies (Bestmann, Baudewig, Siebner, Rothwell, & Frahm, 2004; De Weijer et al., 2014). TMS‐induced activity in the sgACC has only been observed for DLPFC stimulation and not for stimulation of M1. The activation of known motor regions for stimulation of M1, and differences in propagation patterns between two different stimulation sites provides adequate evidence that concurrent single pulse TMS‐fMRI can be used to investigate individual propagation patterns.

### 4.1 Variability in propagation patterns

Although TMS coil placement was well‐controlled and head movement was limited, we observe that TMS‐induced brain activity is variable between participants (Figure 5). More specifically, we observe that stimulation of the same neuroanatomical region results in substantially different propagation patterns of TMS‐evoked activity. An explanation of the variability in the propagation patterns are differences in structural brain connectivity, i.e. differences in propagation pathways, between participants (Bürgel et al., 2006; Thiebaut de Schotten et al., 2011). Another explanation comes from differences in (functional) neuroanatomy (Amunts et al., 1999; Der Malsburg, Phillips, & Singer, 2010; Watson et al., 1993). Stimulation of the same neuroanatomical region does not necessarily mean stimulation of the same functional region. Stimulation of different functional regions can lead to differences in propagation pathways, depending on individual functional connectivity. Finally, functional brain connectivity shows substantial variability between participants, with strong variability observed in the prefrontal cortex (Mueller et al., 2013). Moreover, resting state functional connectivity studies show that brain functional connectivity shows substantial temporal variability, especially in the sgACC (among other regions) (Allen et al., 2014; Handwerker, Roopchansingh, Gonzalez‐Castillo, & Bandettini, 2012; Mueller et al., 2013). To summarize, the structural and functional organization of the brain together with the dynamic nature of functional connectivity could explain the variability in propagation patterns of TMS‐induced activity.

Finally, the complex interaction of the TMS E‐field with the neuronal populations in the cortex has been shown to be brain‐state dependent and therefore not consistent over time (Romei et al., 2008; Sauseng, Klimesch, Gerloff, & Hummel, 2009). EEG recordings have revealed that the pre‐existing neuronal oscillatory activity in the TMS target region affects properties of TMS‐evoked activity. For example, the amplitude of TMS‐induced motor‐evoked potentials depends on pre‐existing oscillatory activity of the primary motor cortex while phosphene thresholds depend on pre‐existing activity in the visual cortex (Romei et al., 2008; Sauseng et al., 2009). Since local neuronal oscillatory activity shows temporal and spatial variability, the TMS effects will also show temporal and spatial variability, which is likely to contribute to the observed variability of propagation patterns of TMS‐induced activity (Allen et al., 2014; Arieli, Sterkin, Grinvald, & Aertsen, 1996; Fox, Corbetta, Snyder, Vincent, & Raichle, 2006).

Our findings are in agreement with prior literature, which indicates that propagation of evoked activity is a complex process that, similar to functional connectivity, varies significantly between individuals (Fox et al., 2006; Handwerker et al., 2012; Mueller et al., 2013; Sauseng et al., 2009). Consequently, the ability of rTMS to modulate specific brain regions, and thus inducing an antidepressant effect, is likely to depend on the state of the individual brain network. Concurrent TMS‐fMRI allows identification of the present individual structural and functional brain organization and connectivity, related to stimulation of a specific area of interest, such as the DLPFC. This concept can potentially be used to predict treatment outcome or to increase treatment efficacy of repetitive stimulation of the DLPFC in MDD. For example, by targeting treatment at the functional region near the DLPFC that leads to individual sgACC activation, assuming the pathway from prefrontal cortex to sgACC is the mechanism of action of the antidepressant effect of rTMS, as has been suggested (Baeken et al., 2014; Fox et al., 2012). In this way, future studies can investigate whether propagation of TMS‐induced activity to sgACC is an accurate predictor of a clinical response to rTMS treatment of the DLPFC in a clinical population of patients with MDD.

We discussed possible (functional) neuroanatomical and state dependent explanations for the variability in TMS‐induced activity between participants. However, the observed variability might originate from other sources. Variability can arise from the physiological interaction between the MRI static magnetic field and the electrophysiology of the individual brain or from the physical interaction between the TMS coil position with respect to the MRI static magnetic field. These complex interactions are subject of extensive research by others and were not directly investigated in this study (Yau, Jalinous, Cantarero, & Desmond, 2014). Future research is required for correct characterization of these interactions and to understand whether they could be responsible for the observed inter‐participant variability.

Finally, TMS‐evoked activity consists of the activity of interest and confounding activity, such as auditory and somatosensory activity induced by the “clicking” sound of the TMS coil and the superficial stimulation of the skin respectively (Lisanby, Gutman, Luber, Schroeder, & Sackeim, 2001). We attempted to minimize confounding brain activity by contrasting TMS pulses of 115% RMT with TMS pulses of 60% RMT. However, a contrast with low intensity TMS does not completely eliminate confounding activity. Consequently, somatosensory and auditory activity should be interpreted with caution.

### 4.2 TMS‐induced activity in the TMS target area

TMS‐induced activity can generally be observed in the TMS target area. However, two participants do not show activity in the vicinity of the TMS coil for stimulation of the left DLPFC. The absence of TMS‐induced activity in the TMS target area has been reported previously (Baudewig et al., 2001; Bestmann et al., 2005; De Weijer et al., 2014). A possible explanation is that in some cases the TMS‐induced currents depolarize the descending white matter tracts, rather than the cell bodies, bypassing synaptic transmission in the TMS target area. As synaptic transmission makes up the majority of the hemodynamic signal measured using fMRI, concurrent TMS‐fMRI is predominantly sensitive to synaptic transmission evoked by TMS (Tagamets & Horwitz, 2001).

### 4.3 Image artifact

The application of concurrent TMS‐fMRI is challenged by numerous technical difficulties, a few of which have already been addressed in other works (Bestmann et al., 2004; Ruff et al., 2008). A technical issue which was not previously described is that we observed short deflections (one sample) in baseline activity in a single slice in the vicinity of the TMS coil in EPI volumes during inspection of the BOLD signal (section “Data analysis”). These artifacts were only observed during sessions in which TMS intensity was changed during the experiment and were absent when the machine output was kept constant. This suggests that currents leaked in the TMS coil, creating a local magnetic field around the TMS coil. This local magnetic field perturbed the MRI static magnetic field during image acquisition, resulting in image distortions. Unfortunately, the cause of the artifact could not be identified with complete certainty.

Fortunately, the effect on the results is negligible, as only a few slices were affected per participant (in <0.5% of all acquired slices per participant) and the artifact in these slices could effectively be removed through interpolation. However, TMS‐induced activity in the vicinity of the TMS coil should be interpreted with caution.

## 5 CONCLUSIONS

In conclusion, TMS pulses delivered to the left DLPFC induce activity in a number of connected brain regions, including the sgACC in some participants (4 out of 9). This indicates that the effects of stimulation of the left DLPFC stimulation have the ability to propagate to the sgACC, depending on individual structural connectivity, providing a potential mechanism for the antidepressant effect of rTMS delivered to the left DLPFC. This individual propensity could potentially be used as a predictor of the response to rTMS treatment in patients with MDD. Additionally, the propagation patterns of TMS‐evoked activity show substantial variability between participants while TMS coil placement was well‐controlled during image acquisition. Combining our observations with prior literature, this implies that the propagation pattern of TMS‐induced activity, and thus the ability of rTMS to modulate specific brain areas, could depend on the current state of the individual brain network.

## Supporting information

Appendix S1: Supporting Information
