# Supplementary material for: A novel concurrent TMS‐fMRI method to reveal propagation patterns of prefrontal magnetic brain stimulation
Source: Hum Brain Mapp. 2018 Aug 29;39(11):4580–92. doi: 10.1002/hbm.24307 (PMC6221049; doi:10.1002/hbm.24307)

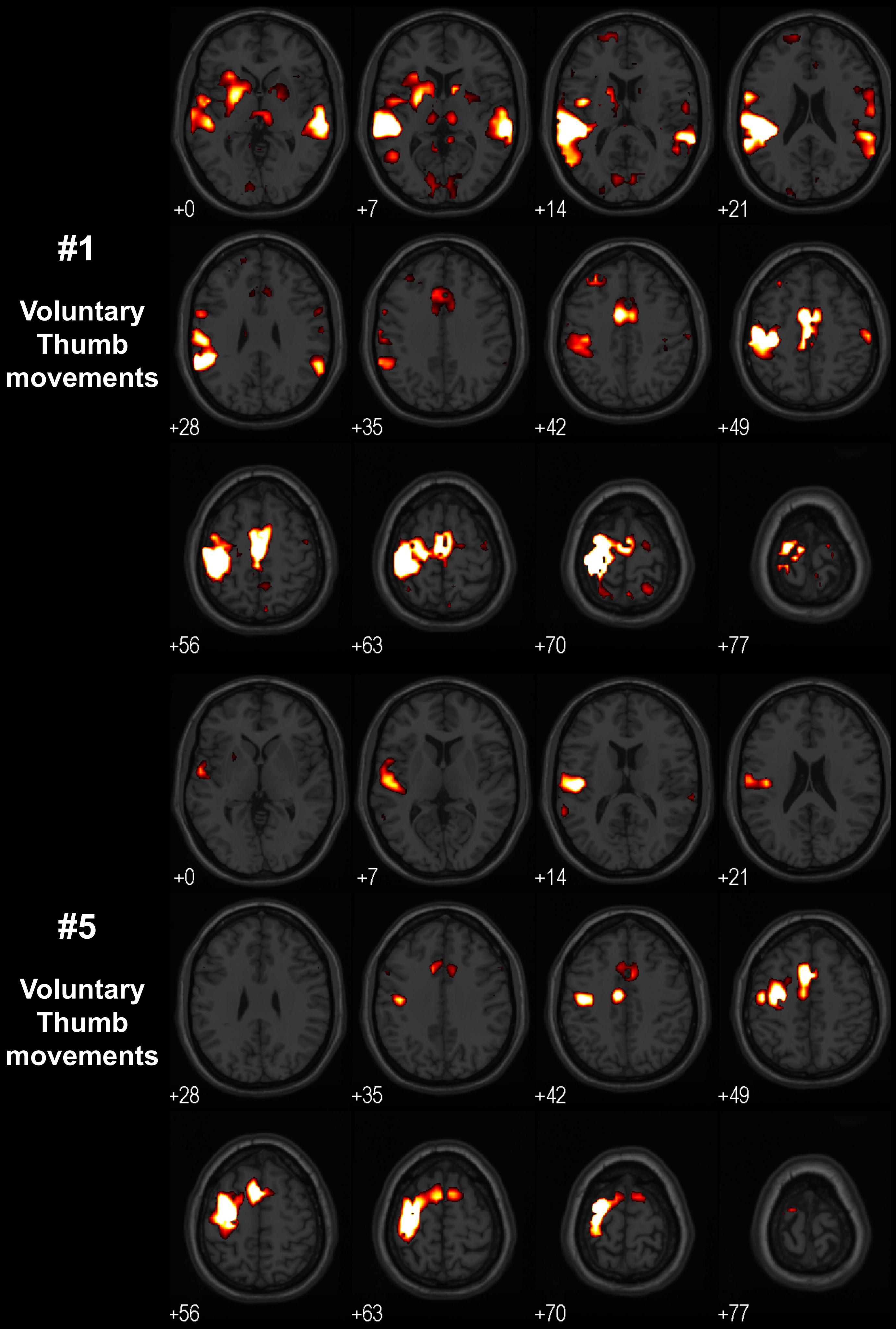

#1
Voluntary Thumb movements
+0
+7
+14
+21
+28
+35
+42
+49
+56
+63
+70
+77
#5
Voluntary Thumb movements
+0
+7
+14
+21
+28
+35
+42
+49
+56
+63
+70
+77

#6

Voluntary Thumb movements

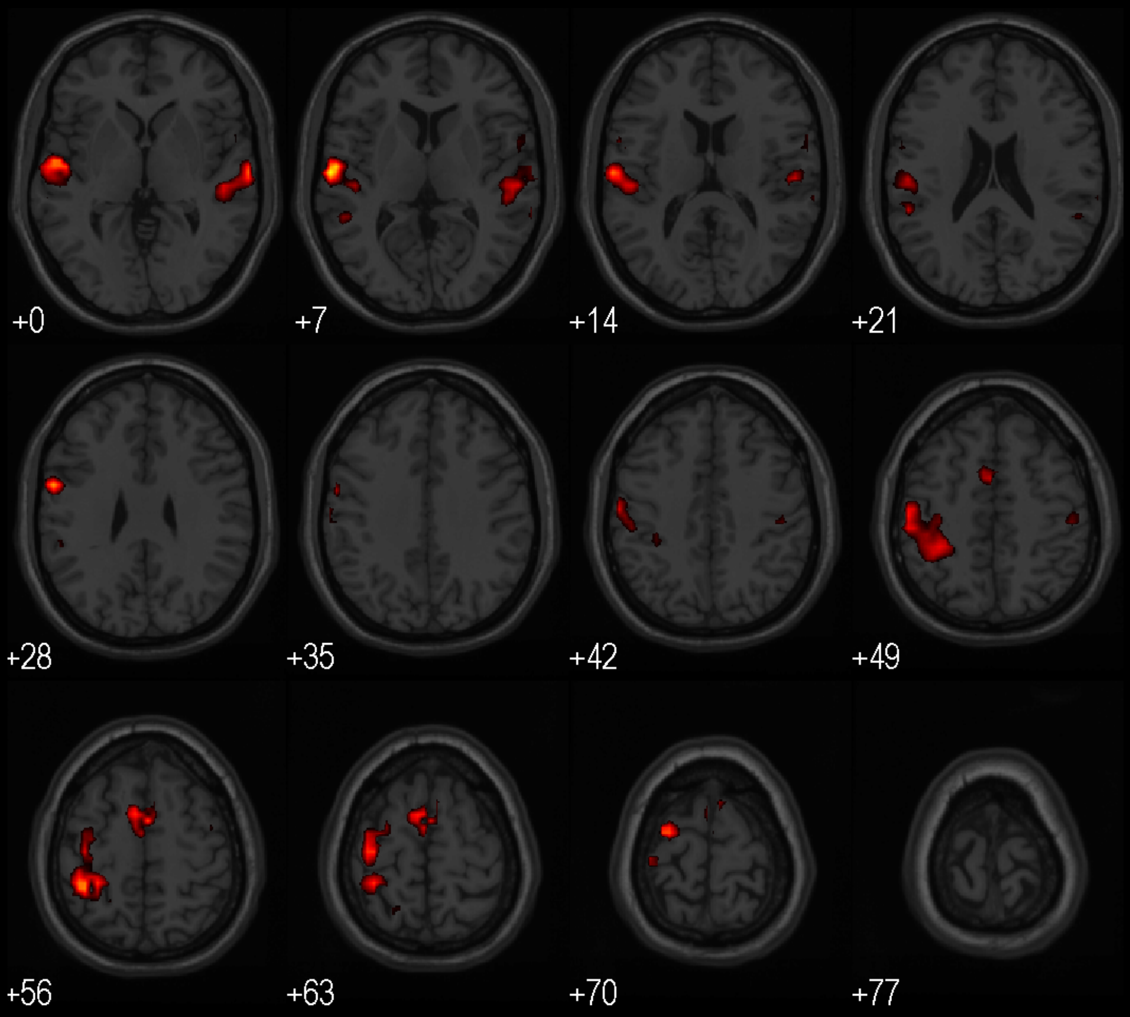

#7

Voluntary Thumb movements

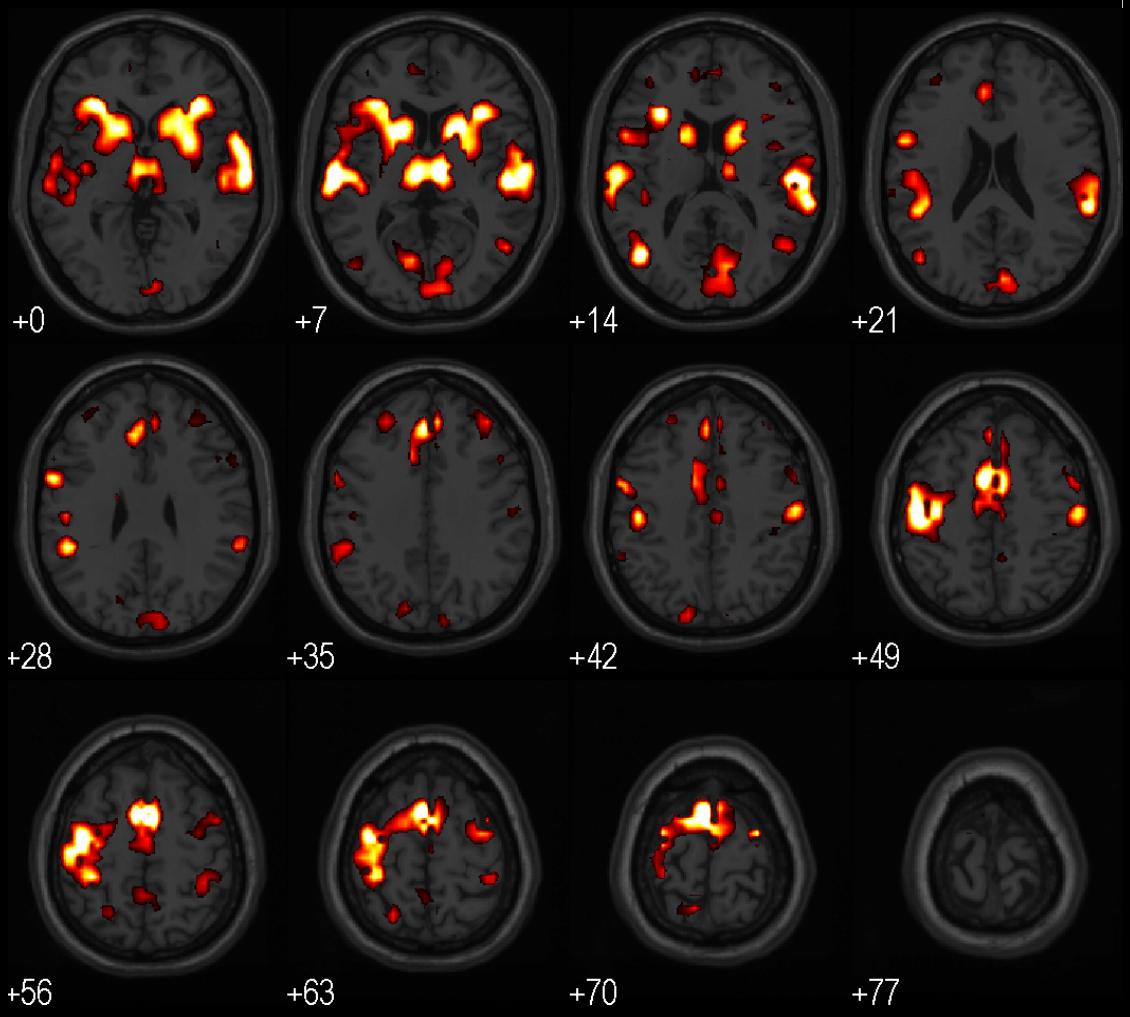

#8

Voluntary Thumb movements

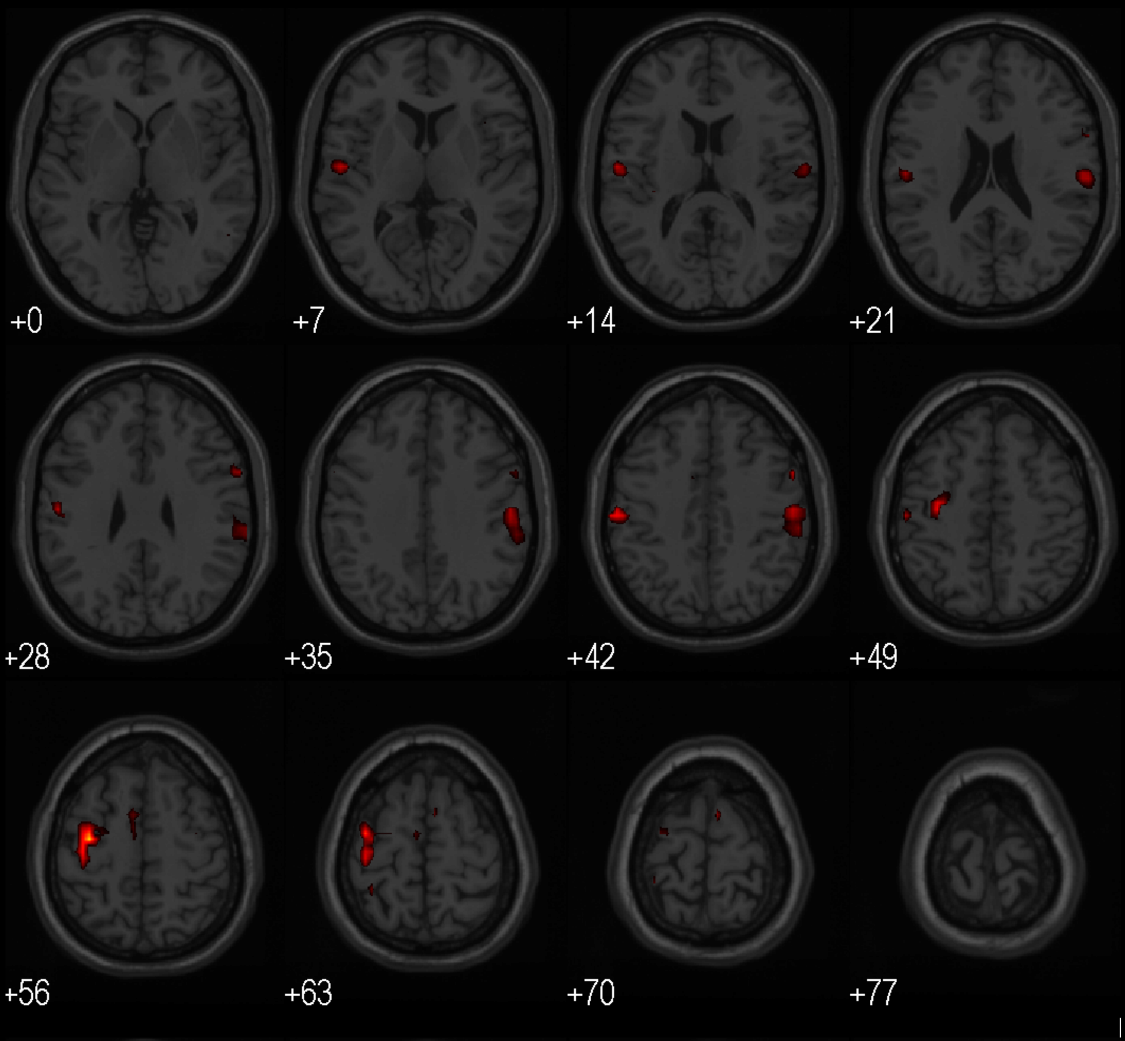

#9

Voluntary Thumb movements

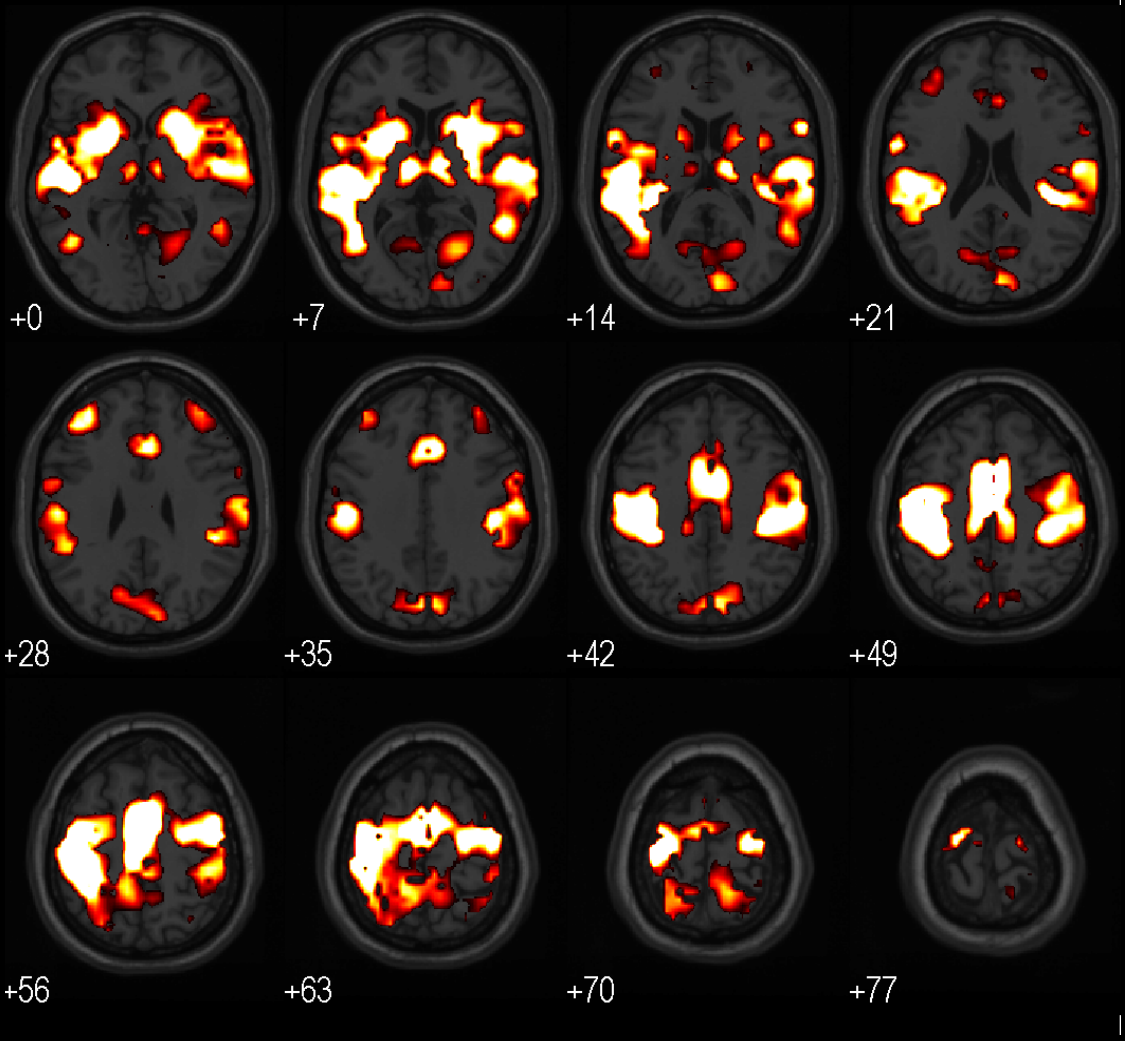

Supplement: Supplementary file 1 — Appendix S1: Supporting Information [file HBM-39-4580-s001.zip › VoluntaryThumbMovements.pdf]
