# Supplementary material for: A novel concurrent TMS‐fMRI method to reveal propagation patterns of prefrontal magnetic brain stimulation
Source: Hum Brain Mapp. 2018 Aug 29;39(11):4580–92. doi: 10.1002/hbm.24307 (PMC6221049; doi:10.1002/hbm.24307)

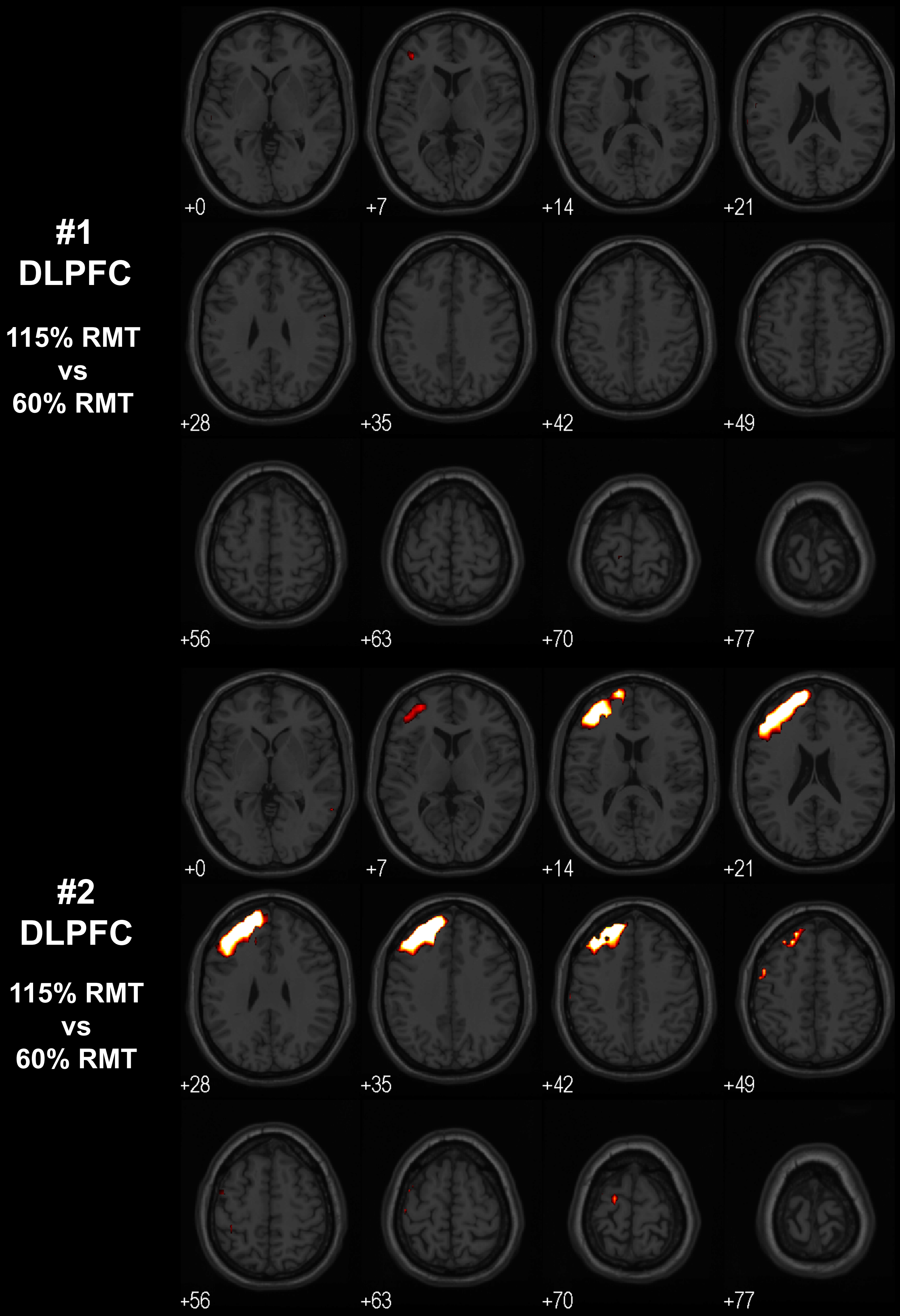
#1
DLPFC
115% RMT
vs
60% RMT
+0
+7
+14
+21
+28
+35
+42
+49
+56
+63
+70
+77
#2
DLPFC
115% RMT
vs
60% RMT
+0
+7
+14
+21
+28
+35
+42
+49
+56
+63
+70
+77

#4
DLPFC
115% RMT
vs
60% RMT

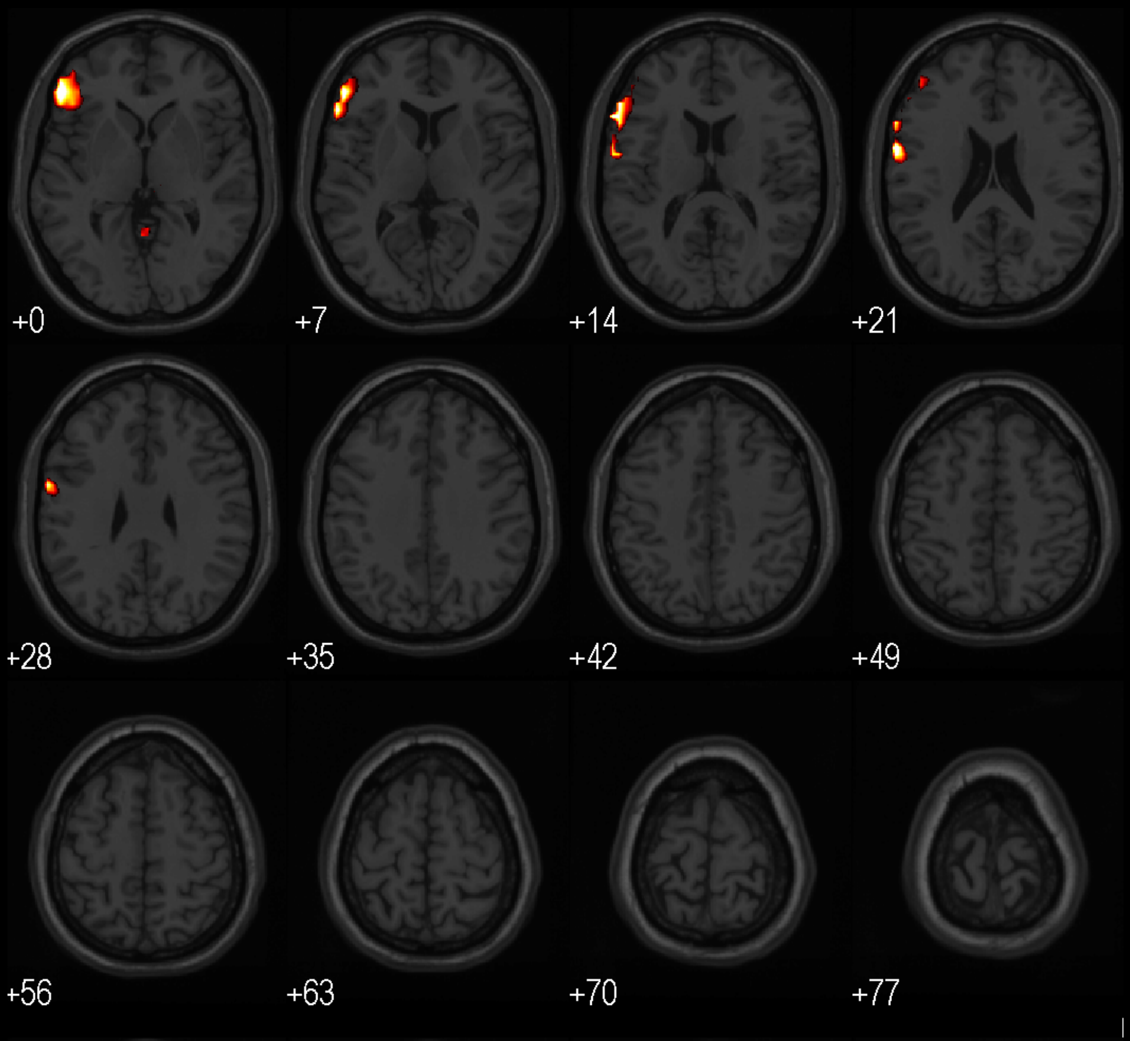

#5
DLPFC
115% RMT
vs
60% RMT

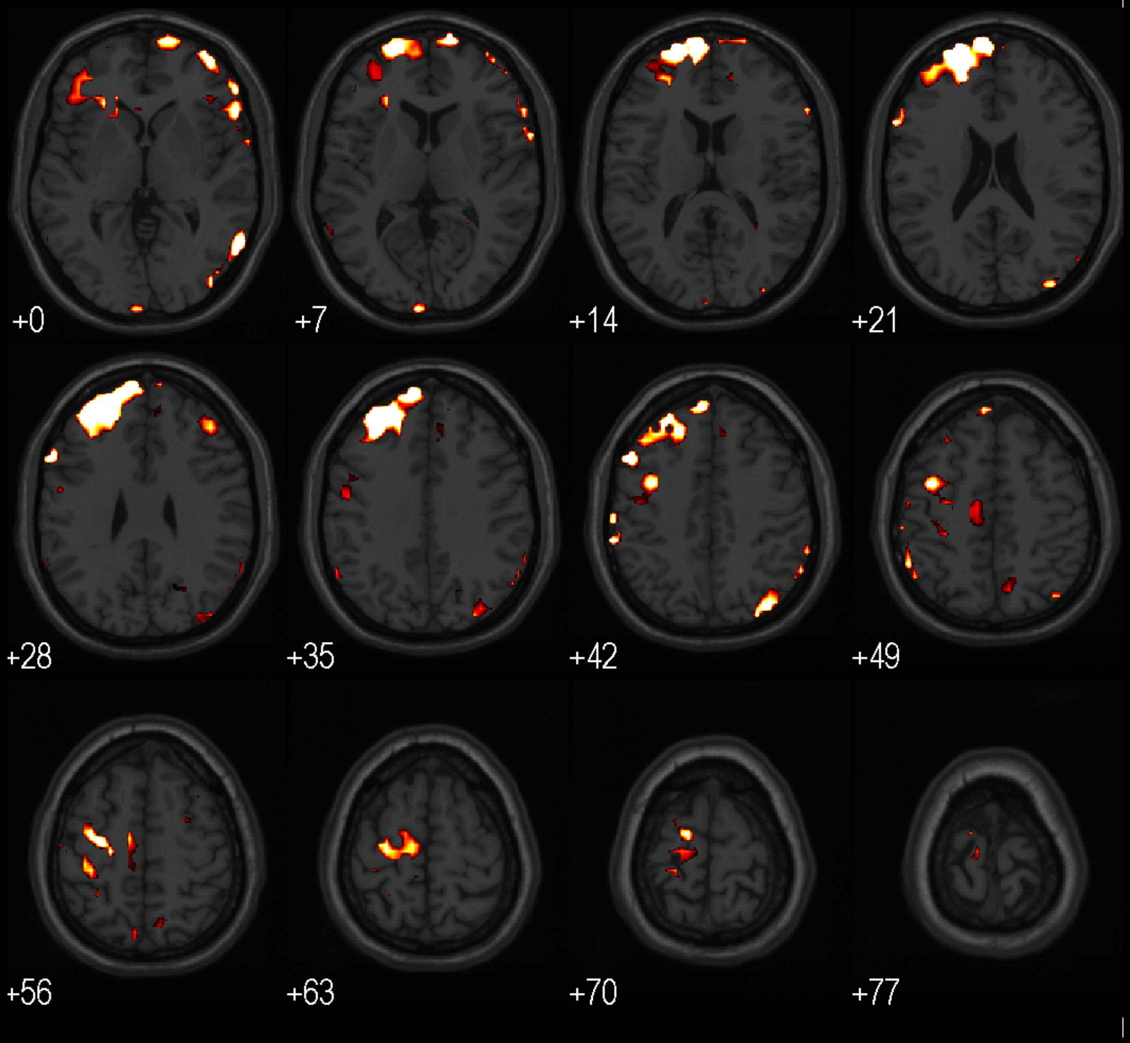

#6
DLPFC

115% RMT
vs
60% RMT

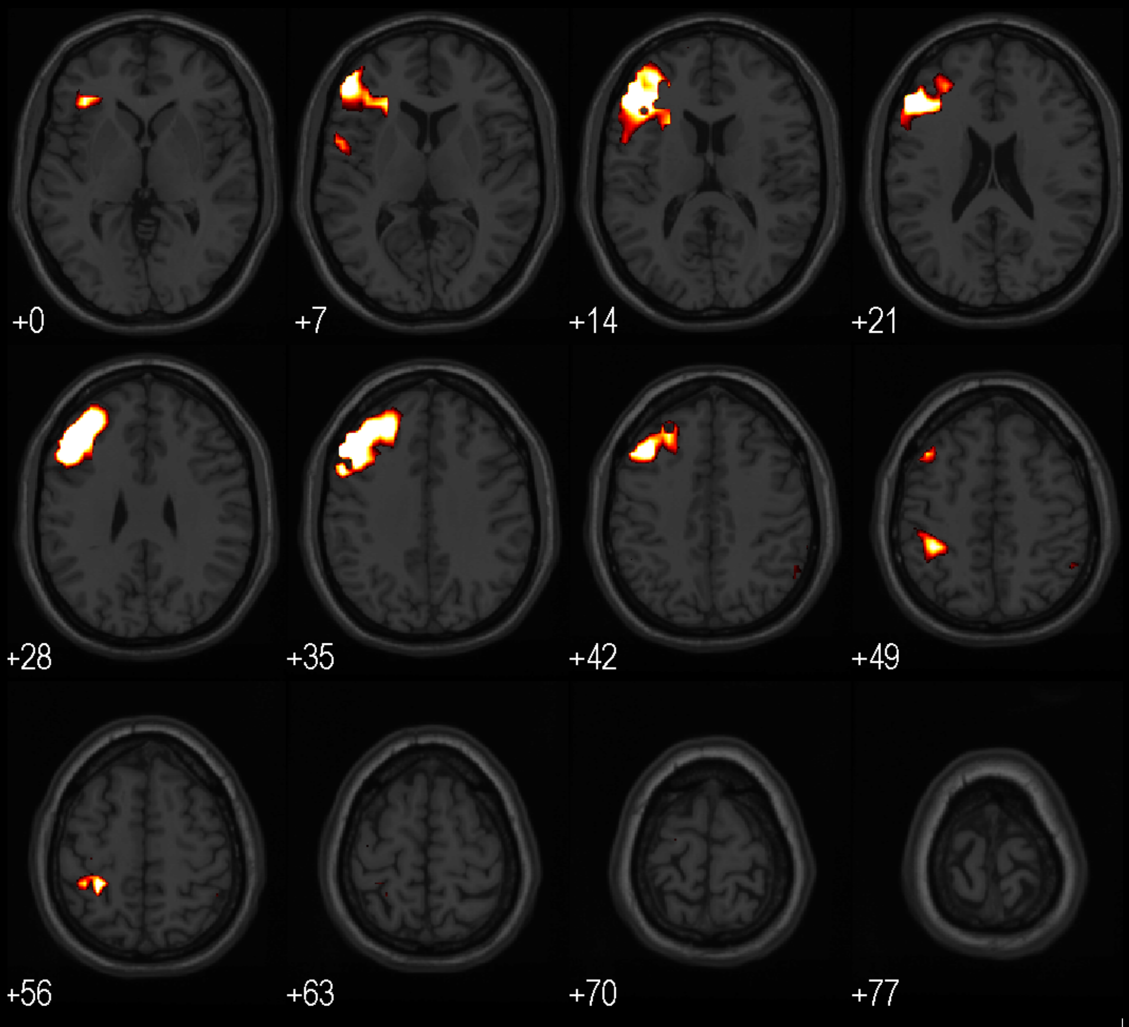

#7
DLPFC

115% RMT
vs
60% RMT

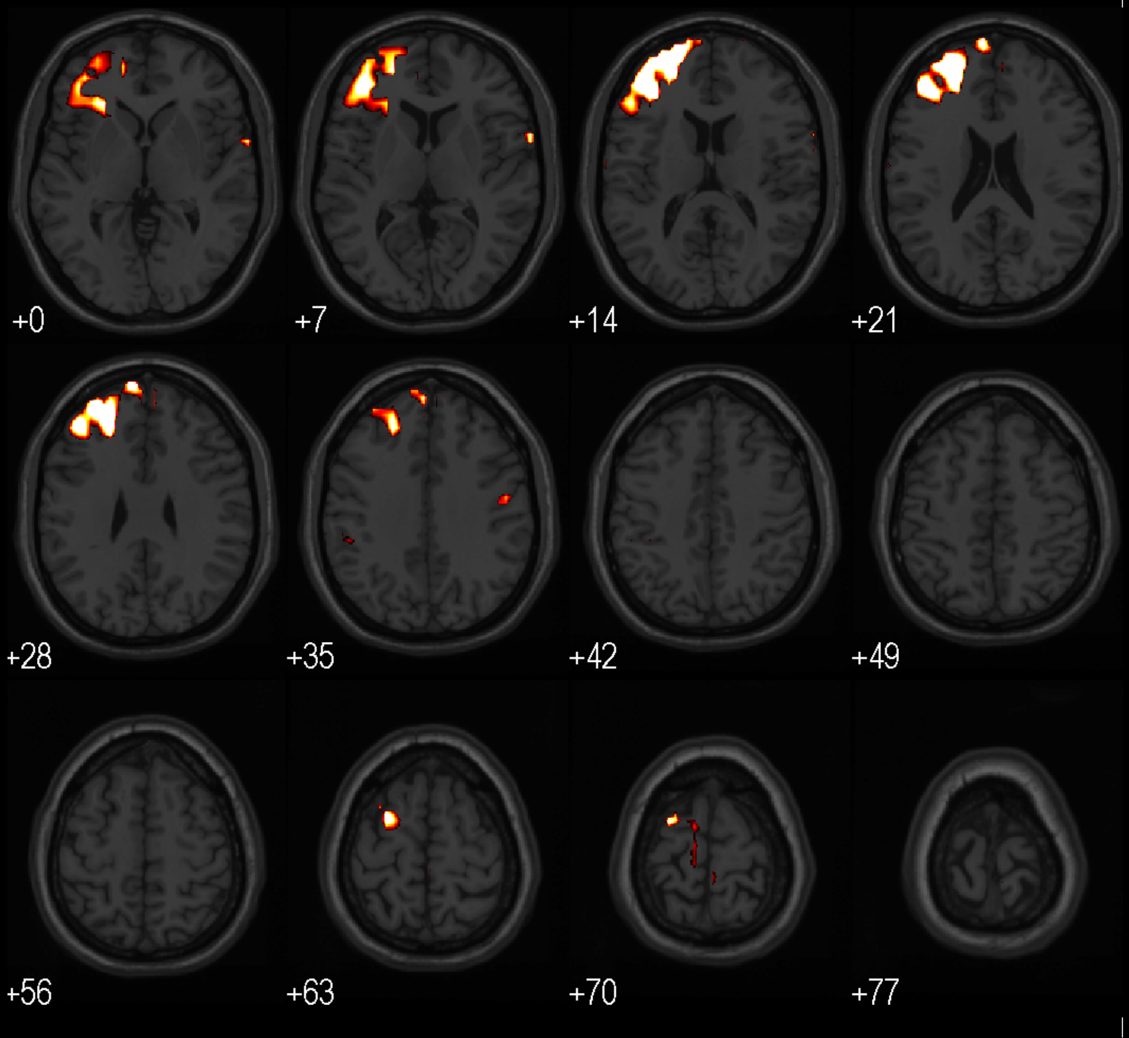

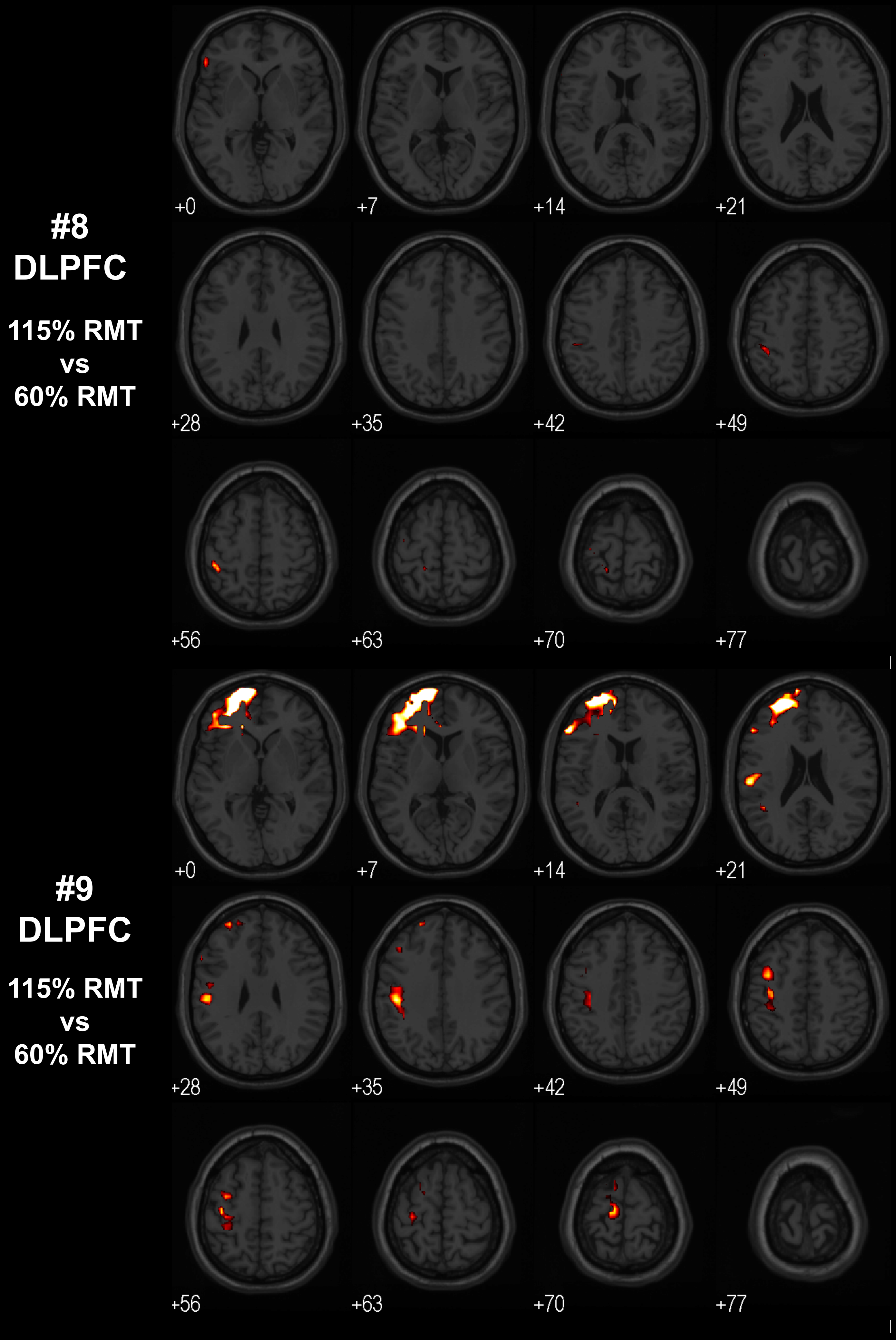
#8
DLPFC
115% RMT
vs
60% RMT
+0
+7
+14
+21
+28
+35
+42
+49
+56
+63
+70
+77
#9
DLPFC
115% RMT
vs
60% RMT
+0
+7
+14
+21
+28
+35
+42
+49
+56
+63
+70
+77

#10
DLPFC

115% RMT
vs
60% RMT

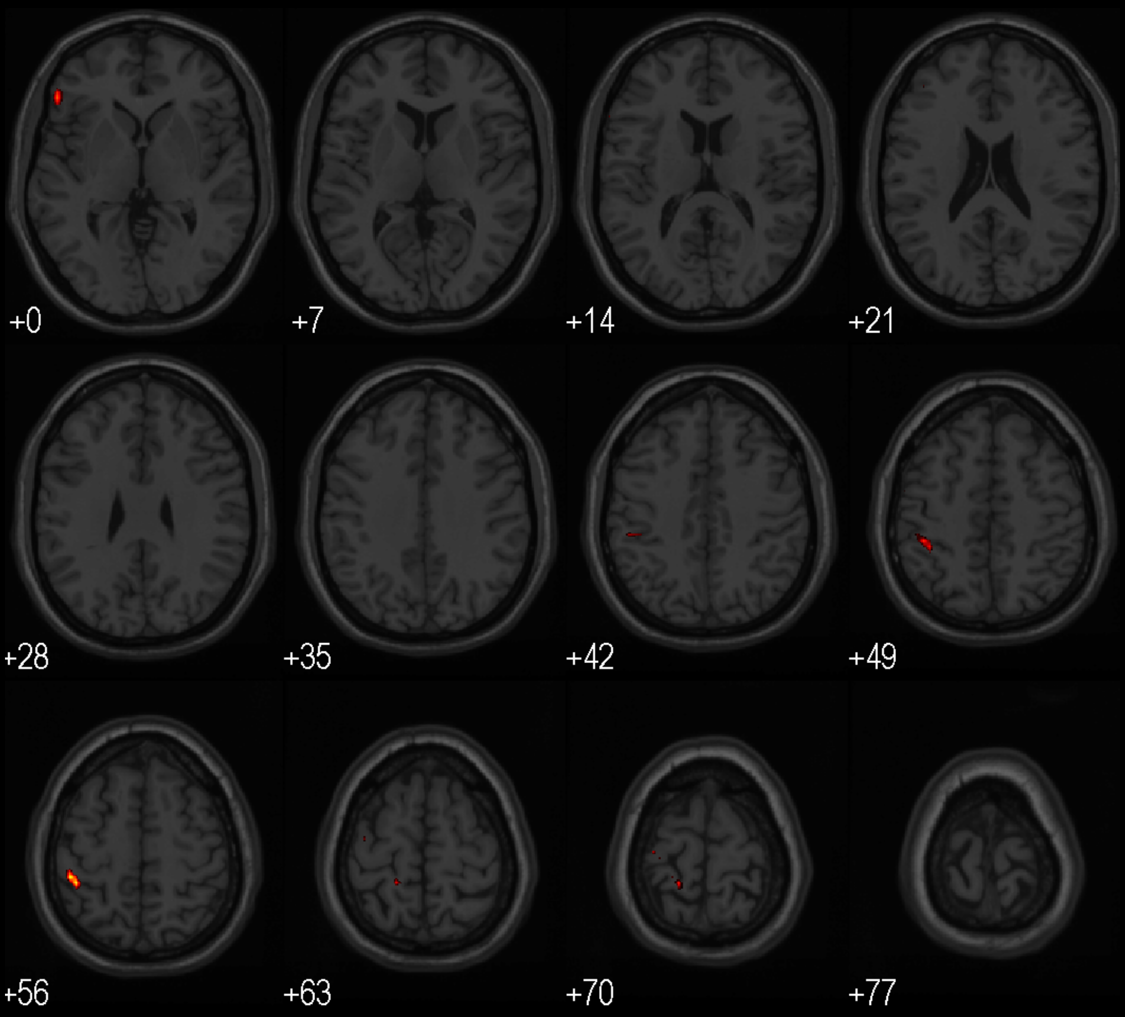

Supplement: Supplementary file 1 — Appendix S1: Supporting Information [file HBM-39-4580-s001.zip › DLPFC_115vs60.pdf]
