# Supplementary figures and images for: A novel concurrent TMS‐fMRI method to reveal propagation patterns of prefrontal magnetic brain stimulation
Source: Hum Brain Mapp. 2018 Aug 29;39(11):4580–92. doi: 10.1002/hbm.24307 (PMC6221049; doi:10.1002/hbm.24307)

**#1**  
**M1**  
**115% RMT**  
**vs**  
**60% RMT**

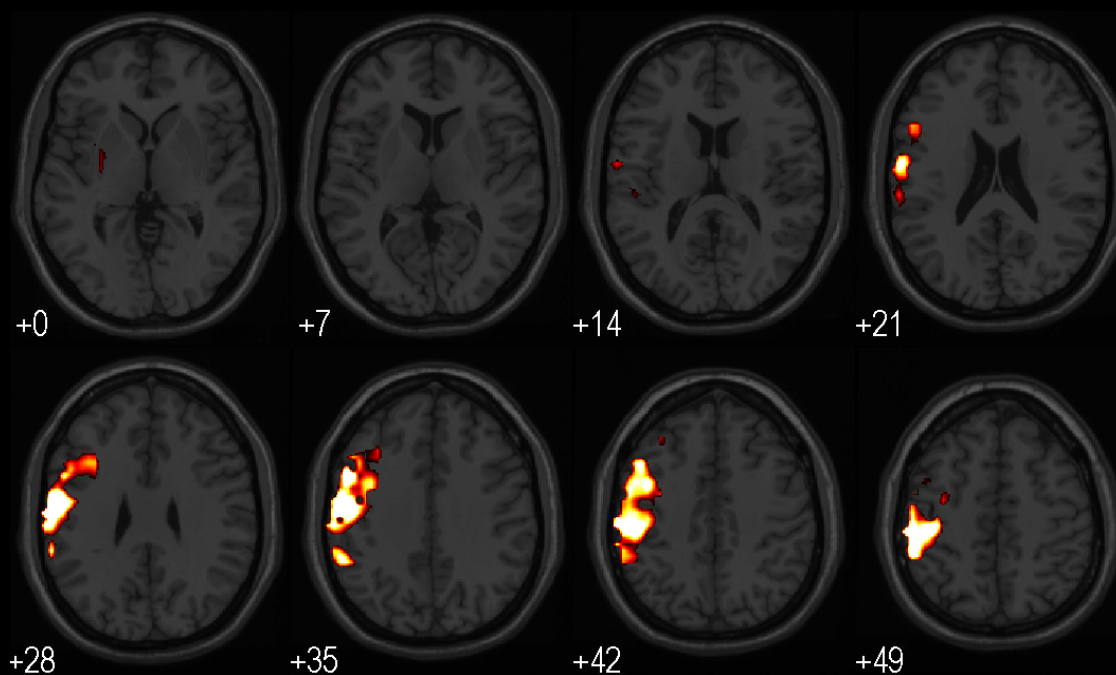

**#5**  
**M1**  
**115% RMT**  
**vs**  
**60% RMT**

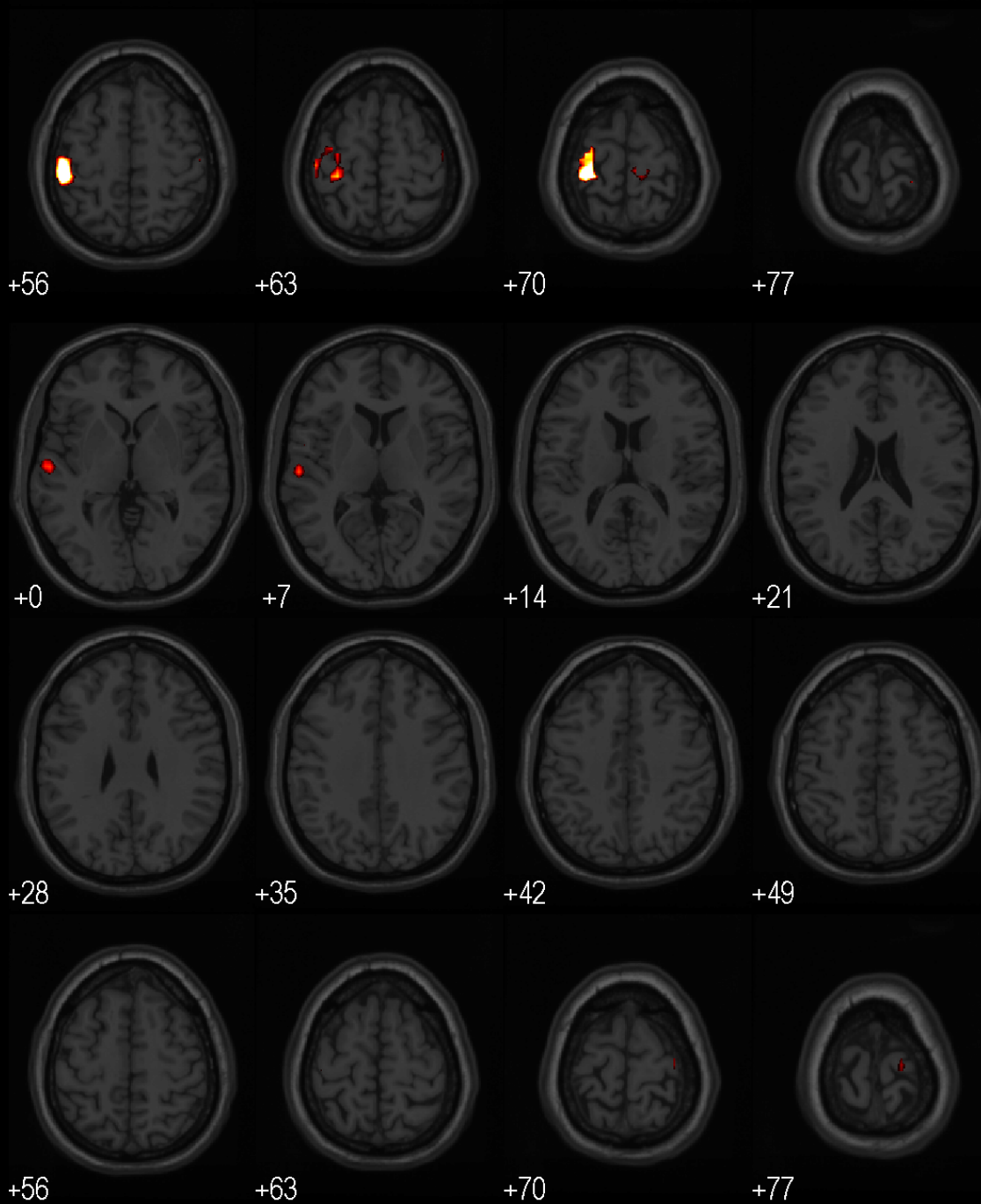

**#6**  
**M1**  
**115% RMT**  
**vs**  
**60% RMT**

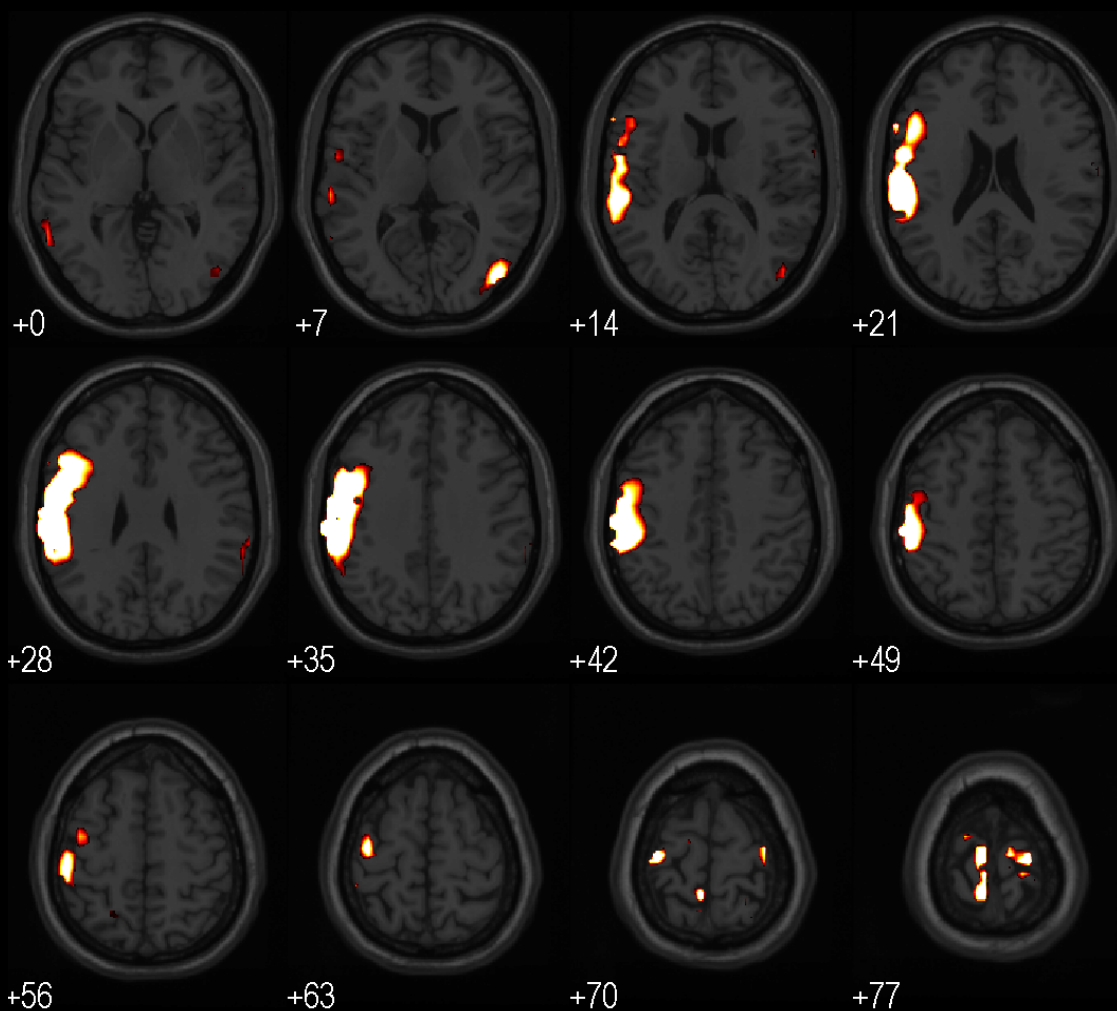

**#7**  
**M1**  
**115% RMT**  
**vs**  
**60% RMT**

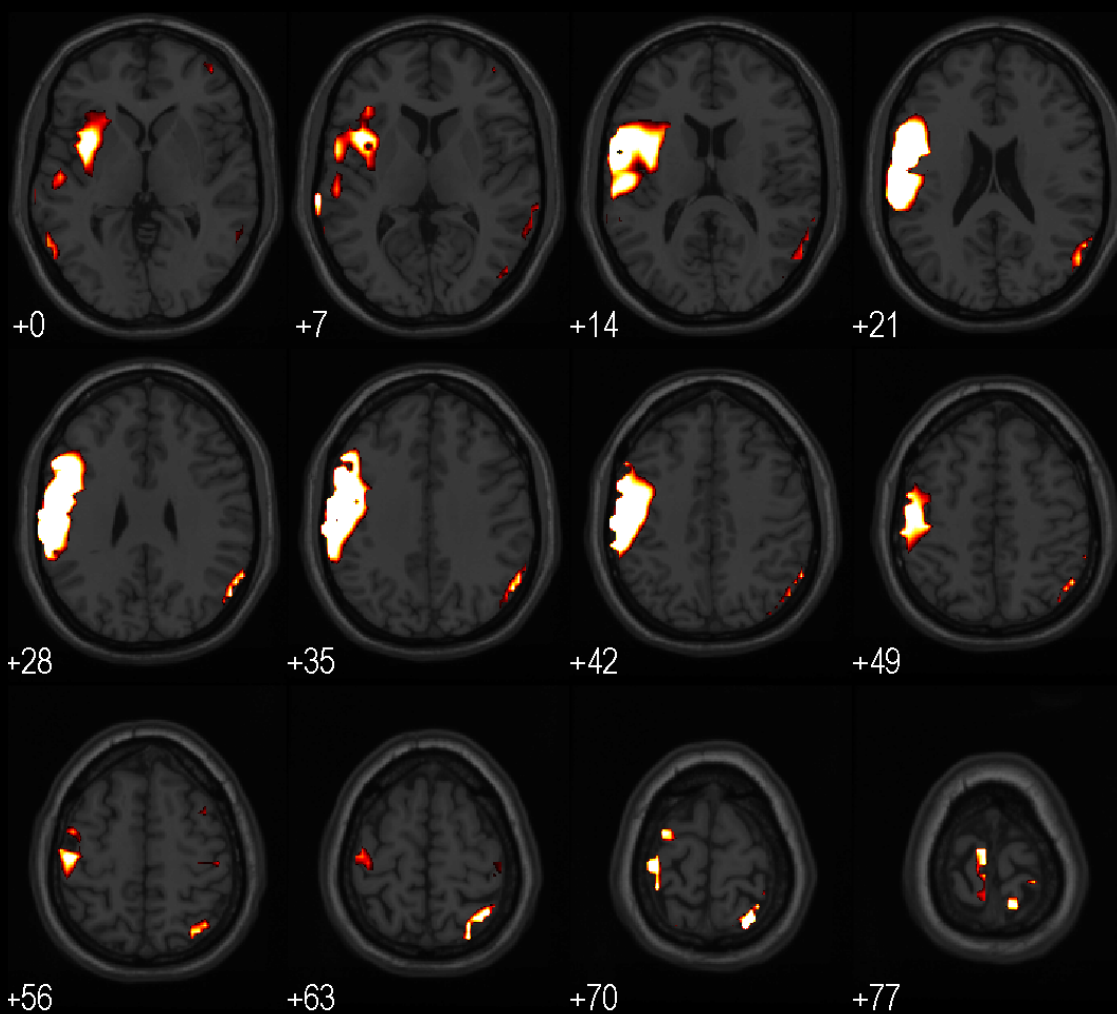

**#8**  
**M1**

**115% RMT**  
**vs**  
**60% RMT**

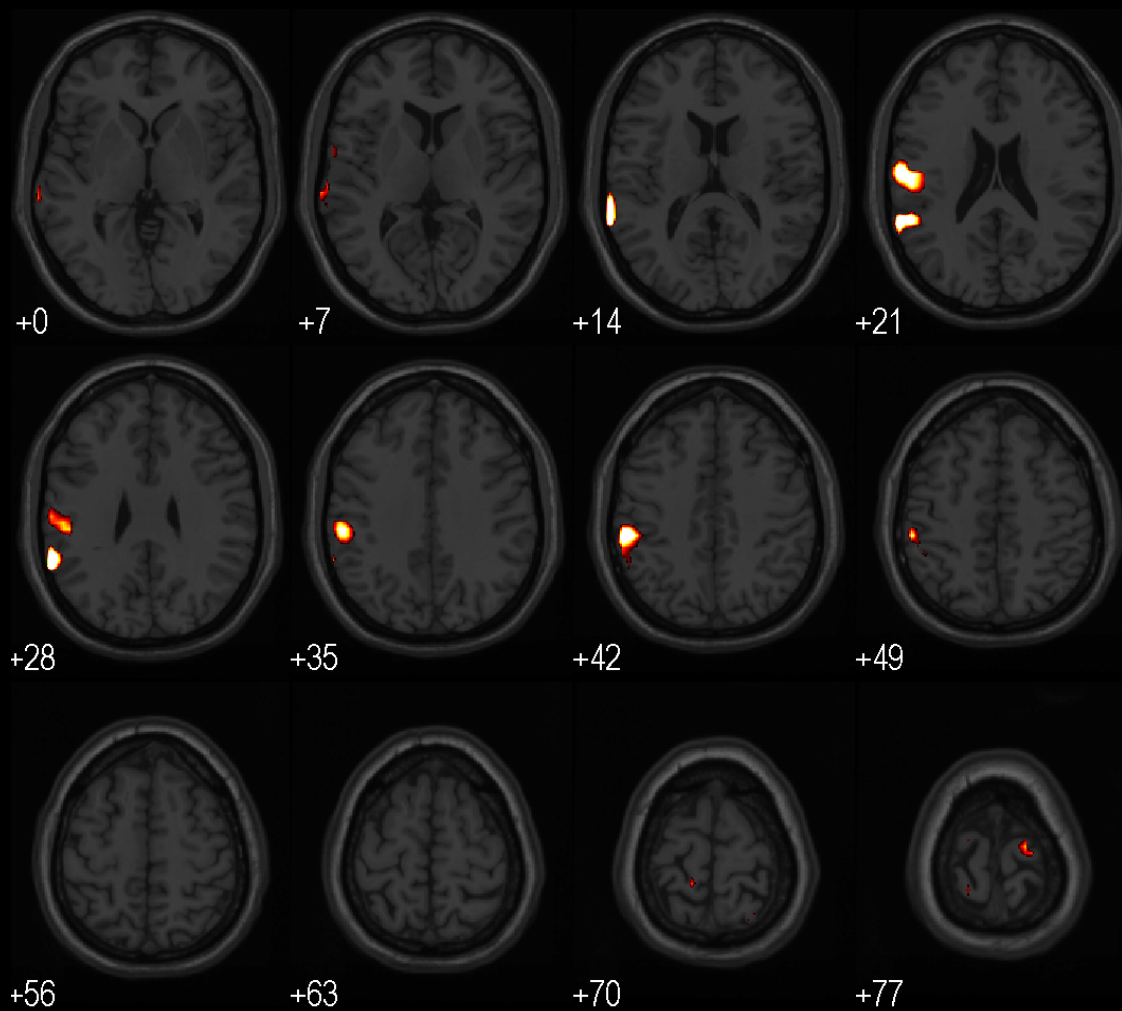

**#9**  
**M1**

**115% RMT**  
**vs**  
**60% RMT**

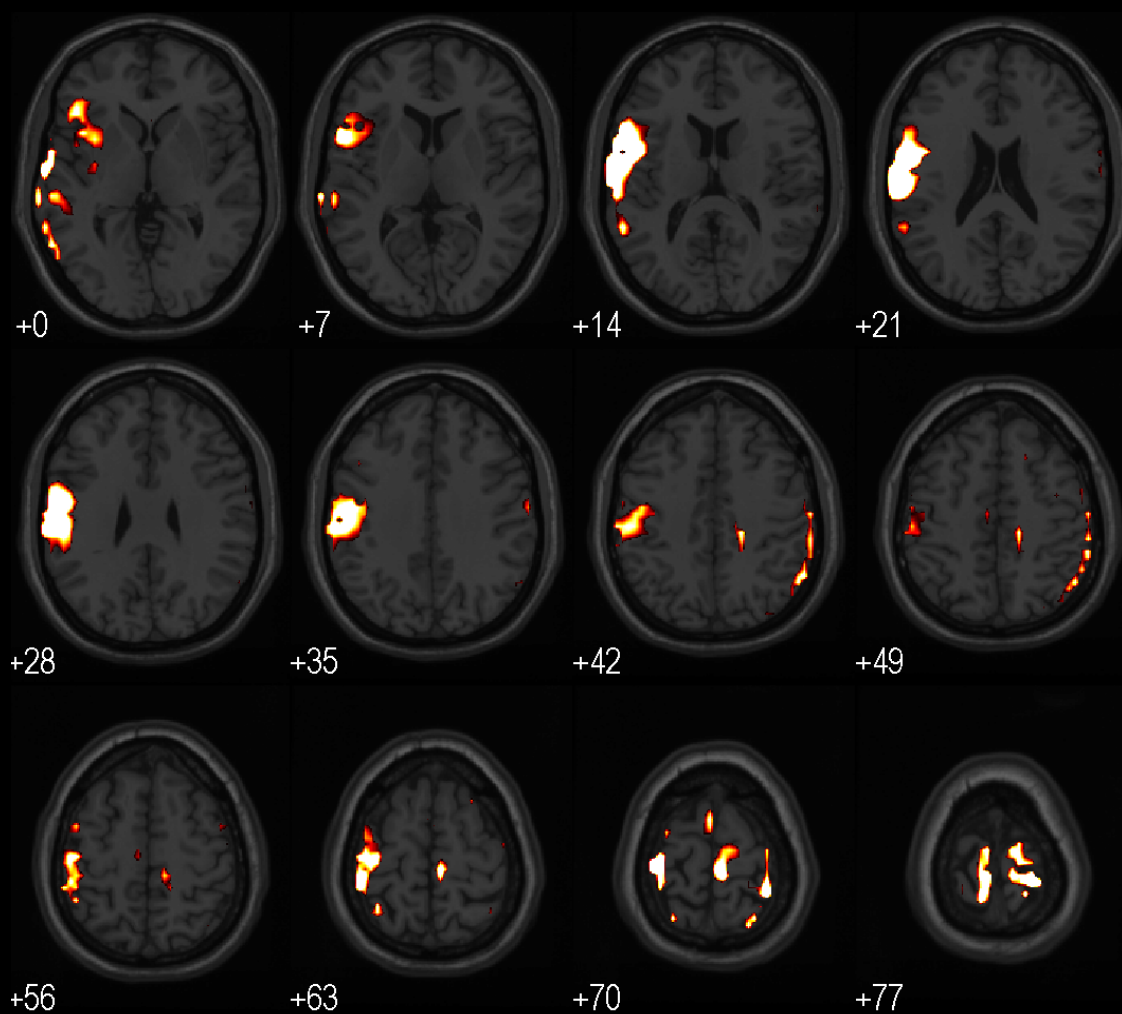

Supplement: Supplementary file 1 — Appendix S1: Supporting Information [file HBM-39-4580-s001.zip › M1_115vs60.pdf]
